# Single‐cell sequencing reveals the role of *IL‐33* ^+^ endothelial subsets in promoting early gastric cancer progression

**DOI:** 10.1002/imt2.70050

**Published:** 2025-06-05

**Authors:** Li Zhou, Mei Yang, Chao Deng, Manqiu Hu, Suhua Wu, Kewen Lai, Lili Zhang, Zhiji Chen, Qin Tang, Qingliang Wang, Lu Chen, Runmin Zha, Yuanyuan Chen, Yibo Tan, Song He, Zhihang Zhou

**Affiliations:** ^1^ Department of Gastroenterology the Second Affiliated Hospital of Chongqing Medical University Chongqing China; ^2^ Department of Gastroenterology the Third People's Hospital of Chengdu Chengdu China; ^3^ Department of Pathology the Second Affiliated Hospital of Chongqing Medical University Chongqing China; ^4^ Department of Ultrasound the Second Affiliated Hospital of Chongqing Medical University Chongqing China

**Keywords:** angiogenesis, early gastric cancer, endothelial cells, *IL‐33*, tumor microenvironment, organoids, single‐cell RNA sequencing

## Abstract

Early gastric cancer (EGC) represents a critical stage in preventing and controlling the progression from gastritis to advanced gastric cancer (AGC). Therefore, identifying the single‐cell characteristics of EGC, particularly the cellular composition of the tumor microenvironment (TME), as well as identifying potential predictive markers and therapeutic targets, could significantly enhance the monitoring of gastric cancer and improve clinical cure rates. We constructed a comprehensive single‐cell RNA sequencing atlas for 184,426 high‐quality gastric cancer cells from various stages, utilizing clinical biopsies and surgical samples. Our single‐cell atlas highlights the cellular and molecular characteristics of EGC. Eight distinct cell lineage states were identified, and it was observed that the number of epithelial cell meta‐clusters gradually decreased, while the number of T&NK, B, plasma, fibroblast, myeloid, and endothelial cells increased with disease progression. Certain epithelial subclusters (metaplastic stem‐like cells (MSCs), pit mucous‐like cells (PMC‐like), proliferating cells), T‐cell subclusters (T_reg_, *CCR7* ^+^ naive, *CH25H* ^+^ CD4^+^, T_EM_ CD8^+^, and *GFPT2* ^+^ CD8^+^ T cells), and endothelial subclusters (*IL‐33* ^+^ Venous‐1 and *AMAMTSL2* ^+^ Artery‐2) were found to be increased in EGC. The Venous‐1 subcluster was found to express high levels of *IL‐33*. Mechanistically, it was revealed that IL‐33 enhances the survival and angiogenesis of endothelial cells by upregulating the expression of adhesion proteins CD34 and PECAM1. Patient‐derived EGC and AGC organoids were subsequently generated, and it was demonstrated that endothelial‐derived IL‐33 promoted the growth of both EGC and AGC organoids *ex vitro* and *in vivo*. Furthermore, IL‐33 was found to increase the expression of KRT17 in EGC organoids. Notably, we also found that high expression of IL‐33 was positively correlated with the depth of invasion and malignancy of EGC. This study provides novel insights into the single‐cell components involved in EGC and reveals the role of the *IL‐33* ^+^ endothelial subcluster in EGC progression.

## Highlights

- Comprehensive single‐cell analysis of early gastric cancer (EGC).
- *CH25H* ^+^ CD4^+^ T cell subclusters are specifically abundant in EGC.
- *IL‐33* ^+^ and *ADAMTSL2* ^+^ endothelial cells subpopulations increased in EGC.
- *IL‐33* ^+^ endothelial cells promote angiogenesis and growth of EGC by regulating adhesion molecule expression.

## Graphical abstract

A single‐cell atlas was established to reveal the molecular characteristics of early gastric cancer (EGC). In combination with organoids, animal models, and clinical samples, it was found that subpopulations such as pit mucous‐like cell (PMC‐like), proliferating cell (PC), *CH25H* ^+^ CD4^+^ T cell, *IL‐33* ^+^ endothelial cell, and *ADAMTSL2* ^+^ endothelial cell are specifically abundant in EGC. Mechanistically, IL‐33 expressed by endothelial cells can upregulate the adhesion molecules PECAM1 and CD34 to facilitate angiogenesis. Meanwhile, *IL‐33* ^+^ endothelial cells can up‐regulate the expression of KRT17 in EGC organoids to promote tumor growth.

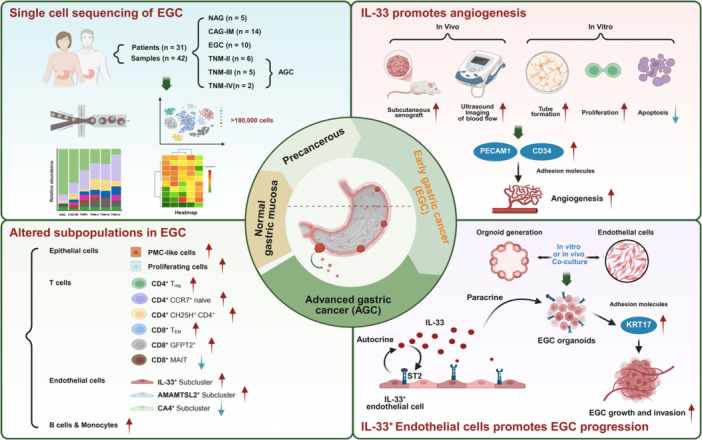

## INTRODUCTION

Gastric cancer (GC) remains one of the most lethal cancers worldwide due to its rapid progression and treatment resistance. Asia has the highest incidence of GC, accounting for >70% of all cases worldwide, and China contributes the most to this burden. Among them, gastric adenocarcinoma accounts for more than 95% of GC [1, 2]. The evolution of gastric cancer involves three stages: first, normal gastric mucosa and non‐atrophic gastritis (NAG) before the initiation of precancerous lesions; second, precancerosis, including chronic atrophic gastritis (CAG), intestinal metaplasia (IM), and intraepithelial neoplasia (IN); and finally, gastric cancer, which can be divided into stages I, II, III, and IV. High‐grade IN and tumor node metastasis (TNM) I GC are identified as early gastric cancer (EGC). More than 80% of gastric cancer patients in China are at an advanced stage when diagnosed, and the overall 5‐year survival rate is less than 30% [3]. Compared with the detrimental prognosis of advanced gastric cancer (AGC), the 5‐year postoperative survival rate of EGC can reach 90%, indicating that the detection and treatment of EGC may be an effective way to prevent tumor progression and improve patient prognosis [4]. However, our understanding of the cellular and molecular characteristics of EGC at the single‐cell level remains poorly understood [5]. Heretofore, only a handful of studies have explored the immune and stromal subtypes of EGC. In 2019, Peng Zhang et al. [6] identified a total of 32,332 high‐quality cells (~2,487 cells per sample) in 13 NAG, CAG, IM, and EGC samples via single‐cell sequencing (scRNA‐seq) technology. Goblet mucosa cells in the IM had an intestinal‐like stem cell phenotype, and several specific markers of early malignant lesions were identified. In 2022, Zhangding Wang et al. [7] obtained a total of 95,551 cells (~6825 cells per sample) from 9 cases of EGC and matched tissues via scRNA‐seq. The discovery of multiple epithelial cell subpopulations that predominate in malignant tissues and the confirmation that *NNMT* ^+^/*AQP5* ^+^ stem cells may contribute to the malignant progression of EGC. However, both have relatively low numbers of high‐quality single cells or insufficient sample sizes. In addition, they explored only the role of specific epithelial cell subsets in IM and EGC. Subsequent studies by Vikrant Kumar [8], Ruiping Wang [9], Jihyun Kim [10], and Ayumu Tsubosaka et al. [11] explored the role of plasma and/or fibroblast subtypes in the progression of IM and GC. They reported that *KLF2* ^+^ epithelial cells recruited by plasma cells in diffuse‐type GC, *IgA* ^+^ plasma cells, and *SDC2* ^+^ carcinoma‐associated fibroblasts (CAFs) are enriched in precancerous lesions and GC, that high expression of epithelial‐myofibroblast transformation is associated with poor clinical prognosis, and that *PDGFRA* ^+^ *BMP4* ^+^ *WNT5A* ^+^ fibroblasts play an important role in the IM. However, these studies focused on AGC rather than EGC and did not confirm the role of other stromal cell subtypes (such as endothelial cells). Cancer cells orchestrate a tumor‐supportive environment by recruiting and reprogramming noncancerous host cells and remodeling the vasculature and extracellular matrix. This dynamic process depends on heterotypic interactions between cancer cells and resident or recruited noncancerous cells of the TME [12].

Angiogenesis, the process of generating new blood vessels, is essential for tumorigenesis [13]. Endothelial cells are the main building blocks of vessels. Tumor endothelial cells exhibit significant heterogeneity and plasticity, controlling the pathways through which proteins, cells, oxygen, and fluids enter surrounding tissues [14]. Endothelial cells that line tumor blood vessels differ from normal endothelial cells. The dysregulated expression of adhesion molecules in tumor endothelial cells can mediate adhesion between tumors and endothelial cells and participate in tumor metastasis and spread [15]. Moreover, they highly express molecules that regulate angiogenesis and permeability, such as vascular endothelial growth factor (VEGF) and angiopoietin [16]. At present, antiangiogenic drugs targeting VEGF/VEGFR have been widely used in tumor therapy [17]. Interleukin‐33 (IL‐33) is predominantly an “alarmin” pro‐inflammatory cytokine belonging to the IL‐1 family of cytokines that respond to various stimuli in endothelial cells, epithelial cells, and fibroblasts [18]. In addition to its pro‐inflammatory effects, *IL‐33* has also been reported to be involved in remodeling the immune microenvironment, influencing angiogenesis, and directly regulating the phenotypes of cancer cells [19, 20, 21, 22]. The binding of IL‐33 to its receptor, ST2, also known as IL‐1 receptor‐like 1 (IL1RL1), is required for its biological activities [23, 24]. In a variety of early solid cancers, IL‐33 is generally highly expressed [25]. It can lead to intestinal metaplasia and malignant transformation of the gastric mucosa [19, 21, 22], induce the development of colorectal adenomas [26], and regulate the progression of esophagitis to esophageal adenocarcinoma [27]. Owing to its important role in the inflammatory response, IL‐33 is expected to be a novel therapeutic target for asthma, cardiovascular diseases, and tumors [28]. Currently, multiple clinical studies on IL‐33 monoclonal antibodies are underway [29]. In gastric cancer, existing studies on IL‐33 have focused mainly on mouse GC models, human‐derived advanced GC cell lines, and clinical samples for *in vivo* simulation and *ex vitro* experiments. The lack of an effective model of EGC has greatly limited the study of its underlying mechanism. The cutting‐edge organoid technology has rapidly progressed, providing a powerful way to study cancers [30].

In this study, we constructed a single‐cell atlas underlying the cellular and molecular characteristics of the gastric epithelial TME across different lesions. We identified 8 distinct cell lineage states and reported that the number of epithelial cell meta‐clusters gradually decreased, whereas the number of T&NK, B, plasma, fibroblast, myeloid, and endothelial cells increased with disease progression. In addition, we found that the proportions of MSCs, PMC‐like, and proliferating cell subclusters increased in EGC; the proportions of T_reg_, *CCR7* ^+^ naive, *CH25H* ^+^ CD4^+^, T_EM_ CD8^+^, and *GFPT2* ^+^ CD8^+^ T cell subclusters increased in EGC; and the proportions of the endothelial cell subpopulations *IL‐33* ^+^ Venous‐1 and *ADAMTSL2* ^+^ Artery‐2 increased in EGC. A panel of EGC‐specific signatures with clinical implications for the diagnosis of EGC was identified. We further found that the number of endothelial Venous‐1 subcluster cells, which highly express *IL‐33*, was increased in EGC. We revealed that IL‐33 could increase the survival and angiogenesis of endothelial cells by upregulating the expression of the adhesion proteins CD34 and PECAM1. We subsequently generated patient‐derived EGC and AGC organoids and revealed that endothelial‐derived IL‐33 promoted the growth of both EGC and AGC organoids *ex vitro* and *in vivo*. IL‐33 increased the expression of KRT17 in EGC organoids. Our study provides novel insights into the single‐cell components of EGC and reveals the role of the *IL‐33* ^+^ endothelial subgroup in EGC progression.

## RESULTS

### A single‐cell atlas of the progression from gastritis to GC

**FIGURE 1:**
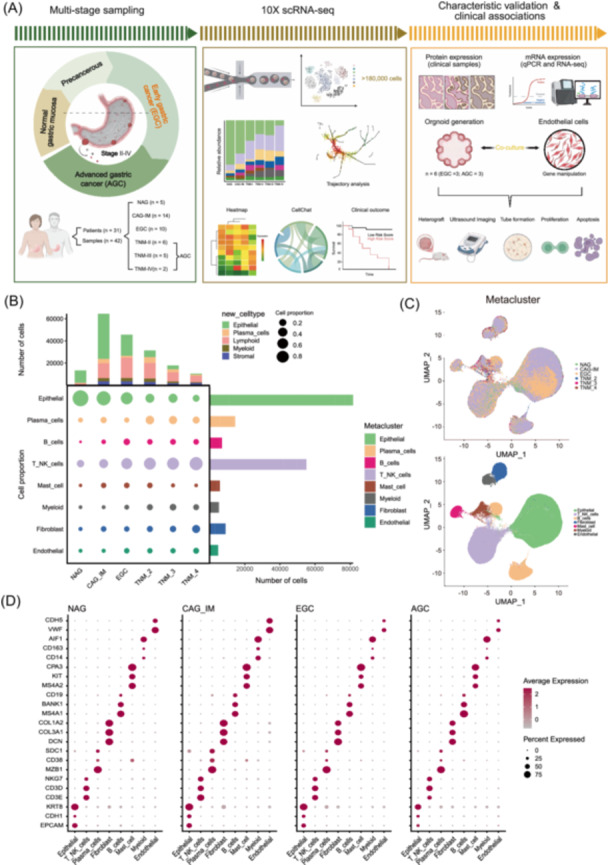
A single‐cell atlas of the progression from gastritis to gastric cancer (GC). (A) Schematic depicting the study design, created with BioRender.com. Thirty‐one patients with gastric cancer undergoing surgical resection or endoscopy had NAG (*n* = 5), CAG‐IM (*n* = 14), EGC (*n* = 10), and AGC (TNM‐II, *n* = 6; TNM‐III, *n* = 5; TNM‐IV, *n* = 2) samples harvested for analysis. scRNA‐seq was performed via the 10× platform, and more than 180,000 cells were sequenced in this study. FFPE tissue blocks from clinical GC patients were subjected to IHC staining, qPCR, and RNA‐seq. Six PDOs (3 EGC and 3 AGC) generated from GC patients were cocultured with HUVECs or their medium supernatant ex vitro and *in vivo*. (B) Cell‐lineage compositions of the progression from gastritis to GC samples inferred from the scRNA‐seq data. Middle (bubble plot), cell subclusters (rows) by stage. The size of the circle represents the cell proportion of each specific cell lineage/type. The circles are color‐coded by defined cell lineages/types, as shown in (C). (C) UMAP of 184,426 cells representing eight unique meta‐clusters. (D) Representative major cell marker genes. The size and color of the circles represent the percentage of cells expressing genes and average gene expression, respectively. The *p*‐value was calculated via a two‐sided Welch's *t*‐test. AGC, advanced gastric cancer; CAG, chronic atrophic gastritis; EGC, early gastric cancer; FFPE, formalin‐fixed paraffin‐embedded; HUVECs, human umbilical vein endothelial cells; IHC, immunohistochemistry; IM, intestinal metaplasia; NAG, nonatrophic gastritis; PDOs, patient‐derived organoids; UMAP, uniform manifold approximation and projection.

Combining the internal and external sample sets, 5 NAG biopsies, 14 CAG‐IM biopsies, 10 EGC samples, 6 TNM‐II samples, 5 TNM‐III samples, and 2 TNM‐IV samples were subjected to single‐cell analysis (Figure 1A, Figure S1, and Tables S1–S3). For each internal sample, we isolated single cells without prior selection for cell types and used droplet‐based scRNA‐seq platforms to generate RNA‐seq data. After low‐quality cells were removed, a total of 184,426 cells (~4391 cells per sample) were retained for subsequent analysis, which yielded a median of 3670 detected genes per sample. The number of cells from each biopsy is provided in Figure 1B and Tables S4 and S5. As shown via uniform manifold approximation and projection (UMAP), profiles along the cascade from NAG and CAG‐IM to EGC and AGC were derived. Eight major cells, referred to as “metaclusters” (epithelial, T/NK, plasma, fibroblast, B, mast, myeloid, and endothelial cells; Figure 1B,C and Figure S2A), and 22 subpopulations, referred to as “subclusters” (Table S6), were finally identified. On the basis of the expression of known markers, we found that the atlas mainly comprised epithelial cells (*EPCAM* and *CDH1*), T/NK cells (*CD3E*, *CD3D*, *NKG7*, and *KLRD1*), plasma cells (*CD27* and *CD38*), fibroblasts (*COL3A1* and *DCN*), B cells (*CD19* and *CD79B*), mast cells (*TPSAB1* and *CPA3*), myeloid cells (*FLT3*, *CD163*, *TPSAB1*, *CD41*, and *CSF3R*), and endothelial cells (*VWF* and *CDH5*) (Figure 1D, Figure S2B, and Table S6). We found that the proportion of epithelial cells decreased gradually from gastritis to AGC. However, the proportions of T&NK cells, B cells, plasma cells, fibroblasts, myeloid cells, and endothelial cells increased following the progression of GC (Figure 1B, Figure S2C,D, and Tables S7–11). These findings are consistent with the clinical development of gastric cancer [31].

### The number of PMC‐like and proliferating cell subclusters among epithelial cells increased in EGC

**FIGURE 2:**
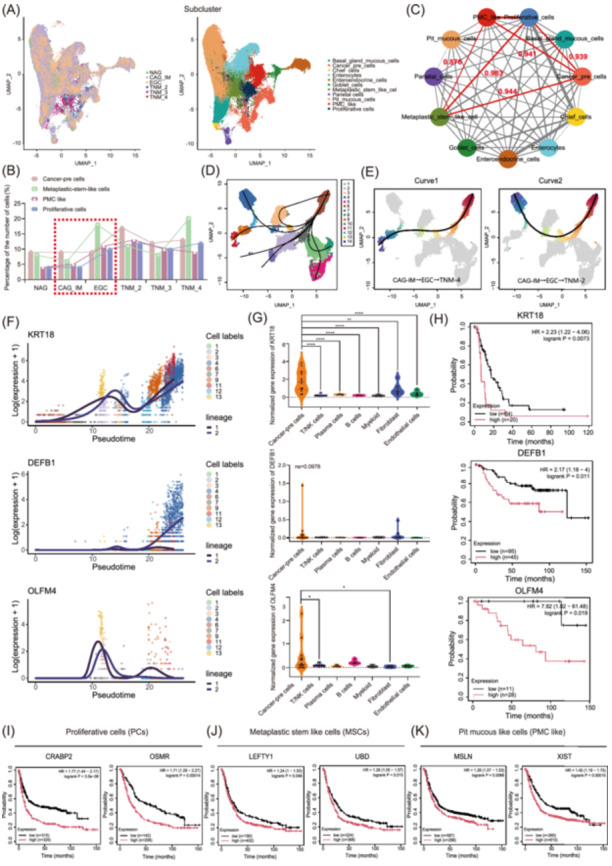
Cell lineage annotation of epithelial cells in GC. (A) UMAP showing the 11 major epithelial cell subclusters identified via scRNA‐seq. (B) The proportions of four representative epithelial cell subsets (cancer‐pre, MSCs, PMC‐like, and PCs) across tissue groups. (C) Similarity network among diverse epithelial cell types in our data set. The thickness of the edges in the network was denoted as the Pearson correlation coefficient between the centroids of any pair of cell types. (D, E) Slingshot trajectory analysis of cancer‐pre cells generated via single‐cell experiments. Slingshot trajectory analysis demonstrating differentiation and pseudotime changes in cancer‐pre subsets (Curve1–7). Each black curve represents an independent differentiation lineage in order of inferred pseudotime values (D). Display of individual differentiation lineages. pseudotime values are drawn for each lineage to infer results. The dots in E represent cells, the black lines represent cell differentiation tracks, the color from red to blue indicates pseudotime from early to late, and the gray part of cells indicates that they do not belong to this lineage. (F) Expression dynamics of representative genes (*KRT18*, *DEFB1*, and *OLFM4*) in different tissues (color coded) over pseudotime. The two lines represent the two differentiation lineages, the abscis are the pseudotime values from early to late, and the ordinates are the expression values of the target genes. Each dot represents a cell, and the color represents the cell subgroup to which each cell belongs. (G) Violin expression plot of representative genes (*KRT18*, *DEFB1*, and *OLFM4*) in meta‐clusters. (H) Survival analysis (overall survival, OS) of representative genes (*KRT18*, *DEFB1*, and *OLFM4*) in GC. (I–K) Survival analysis (overall survival, OS) of marker genes in subsets of PCs, MSCs and PMC like. *p*‐values were calculated by one‐way Kruskal‒Wallis rank‒sum tests. *p < 0.05; **p < 0.01; ***p < 0.001; ****p < 0.0001. DEGs, differentially expressed genes; MSCs, metaplastic stem‐like cells; PMC like, pit mucous cells like; PCs, proliferative cells; UMAP, uniform manifold approximation and projection.

For epithelial cell meta‐clusters, we performed UMAP dimensionality reduction cluster analysis again and identified 11 subclusters: basal gland mucous cells (BMCs) (marked by *MUC6* and *TFF2*), cheif cells (marked by *LIPF*, *PGA3*, and *PGA4*), enterocytes (marked by *FABP1* and *APOA1*), enteroendocrine cells (marked by *CHGA* and *CHGB*), goblet cells (marked by *MUC2* and *ITLN1*), MSCs (marked by *OLFM4*, *EPHB2*, and *SOX9*), parietal cells (marked by *ATP4A* and *ATP4B*), pit mucous cells (PMCs) (marked by *MUC5AC* and *TFF1*), pit mucous‐like cells (PMC‐like) (marked by *SOX4*), and proliferative cells (PCs) (marked by *MKI67*) (Figure 2A, Figure S3A, and Table S6), and cancer‐pre cells. We identified one cluster as the cancer‐pre cells, since it was enriched in the EGC biopsy and expressed cancer markers (*CEACAM5* and *CEACAM6*) (Figure 2B and Figure S3B). Moreover, the number of copy number variations (CNVs) in the cancer‐pre subcluster was greater than that in the other subclusters (Figure S3C). Through correlation calculations, we found that there was a significant correlation between MSCs (*R* = 0.944), PMCs (*R* = 0.941), PCs (*R* = 0.939), and cancer‐pre subclusters (Figure 2C and Table S12). We then assessed the cellular heterogeneity of MSCs, PMCs, PCs, and cancer‐pre cells. For the cancer‐pre subcluster, we used slingshot analysis to infer the cell lineage differentiation structure and order and identified seven distinct lineages (Figure 2D and Figure S3D). Curve 1 and Curve 2 differentiation trajectories cover the lineage changes from CAG‐IM to AGC (Figure 2E). With the progression of TNM‐IV GC, the expression of some genes changed (Figure S3E,F). Typically, *KRT18* (Curve1 and Curve2) and *DEFB1* (Curve2) increased gradually from CAG‐IM to AGC. In particular, we also found that *OLFM4* expression increased significantly in EGC and decreased sharply with increasing stage of GC (Figure 2F, Figure S3E, and Table S13). The expression of *KRT18* (*p* < 0.0001) and OLFM4 (*p* = 0.0435) in the cancer‐pre subcluster was significantly greater than that in the other subclusters. Although there was no statistically significant difference in the expression of *DEFB1* (*p* = 0.0978), a difference could still be observed (Figure 2G). We subsequently performed a survival analysis of the above genes in the TCGA database stomach adenocarcinoma (STAD). High expression of *KRT18* ((Overall surviva (OS), *p* = 0.0073)), *DEFB1* (OS, *p* = 0.011), and *OLFM4/OlfD* (OS, *p* = 0.019) was positively correlated with poor prognosis of TNM‐IV and EGC patients, respectively (Figure 2H). These results indicate that with the initiation and development of GC, cancer‐pre cells exhibit diverse differentiation trajectories and express different genes involved in malignant transformation. Gene Ontology (GO) and Kyoto Encyclopedia of Genes and Genomes (KEGG) analyses (Figure S3G,H, and Table S13) revealed that the top upregulated genes (start vs. end) in cancer‐pre cells (Curve1 and Curve2) were enriched in antigen processing and presentation pathways, such as *CD74* [32], *HLA‐DRB1* [33], *HLA‐DRA* [34], *PSME1/2* [35], and *CTSB* [36]. These genes are reported to regulate immune cell infiltration and activation in GC and are correlated with poor prognosis in patients with GC.

PCs (*CRABP2* ^+^, *OSMR* ^+^, and other PCs) and MSCs (C1–C11) were identified into 3 and 11 cell clusters, respectively (Figure S4A–D). In addition to canonical cell type markers, we also identified additional genes that strongly and specifically marked those clusters (Table S6). For example, the GC oncogenes *CRABP2* [37], *LGR6* [38], and *SPON2* [39] were simultaneously highly expressed in *CRABP2* ^+^ (Figure S4E) and C3 (MSCs subcluster; Figure S4E) strains. Moreover, those clusters had a significantly increased proportion of AGC (Figure S4B,D). There are also some specific genes in MSCs that can act as markers for clusters in different stages (Figure S4F). For example, *LEFTY1* is highly expressed in CAG‐IM (Figure S4F), which is consistent with published studies [6]. By using TCGA database analysis, we found that high expression of *CRABP2* (OS, *p* = 3.5e‐08; PFS, *p* = 8.8e‐13), *OSMR* (OS, *p* = 0.00014; PFS, *p* = 0.00031) (PCs, Figure 2I and Figure S4G), *LEFTY1* (OS, *p* = 0.048), *UBD* (OS, *p* = 0.015) (MSCs, Figure 2J), *MSLN* (OS, *p* = 0.0066), and *XIST* (OS, *p* = 0.00015) (PMC‐like, Figure 2K) was associated with poor prognosis in GC patients.

Six PMC‐like clusters were identified (*PLGC2* ^+^ C1, *LIPF* ^+^ C2, *REG4* ^+^ C3, *GAST* ^+^ C4, *MSLN* ^+^ C5, and *XIST* ^+^ C6) (Figure S4H,I). The *LIPF* ^+^ C2 subcluster accounted for the majority of NAG samples, whereas the *MSLN* ^+^ C5 and *XIST* ^+^ C6 subclusters gradually increased during the transition to EGC and further AGC (Figure S4J). The XIST^+^ C6 subcluster accounted for the majority of the TNM‐IV samples. By analyzing the differentially expressed genes (DEGs) (Figure S4K) and KEGG pathway analysis (Figure S4L) of the C5/C6 and C2 subclusters, respectively, we found that the DEGs in both the *MSLN* ^+^ C5 and the *XIST* ^+^ C6 subclusters were involved mainly in the ribosome pathway. Kaplan‐Meier survival analysis (Figure S4M) revealed that the marker genes *PLCG2* (C1, OS, *p* = 0.026), *LIPF* (C2, OS, *p* = 0.1), *REG4* (C3, OS, *p* = 0.065), and *GAST* (C4, OS, *p* = 0.021) were closely related to the prognosis of patients with GC. In conclusion, among epithelial cells, the number of cancer‐pre, MSC, PMC‐like, and PC subclusters increased in EGC, revealing the differentiation trajectories among cancer‐pre subclusters.

### T_reg_, *CCR7* ^+^ naive, *CH25H* ^+^ CD4^+^, T_EM_ CD8^+^, and *GFPT2* ^+^ CD8^+^ T cells increased, whereas MAIT CD8^+^ T cells decreased in EGC

Understanding the complex interplay between tumor cell‐intrinsic, cell‐extrinsic, and systemic mediators of GC progression is critical for its treatment. We examined the immune and stromal cellular abundances and compositions of major lineages during EGC progression. The proportions of T&NK (*CD3E* and *CD3D*), myeloid (*ITGAM* and *CD11b*), B cells (*CD19* and *CD79B*), plasma cells (*CD27* and *CD38*), fibroblasts (*DCN* and *COL3A1*), and endothelial cells (*VWF* and *CDH5*) increased significantly during GC progression (Figure 1A).

**FIGURE 3:**
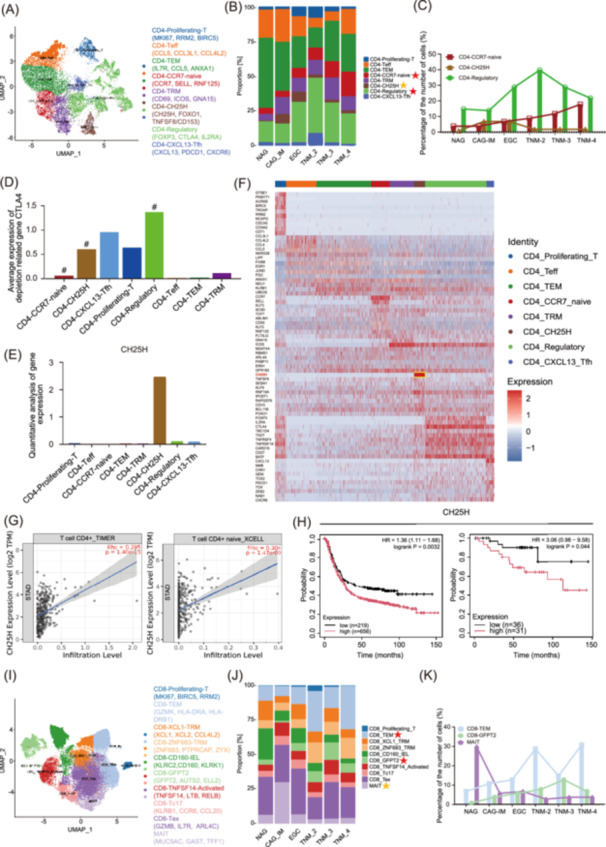
Characterization of T cell states. (A) UMAP view of 8 CD4^+^ T cell clusters. (B) Proportions of each subset in the CD4^+^ T cluster. (C) Proportions of three representative CD4^+^ T cell sub‐populations (*CCR7* ^+^ naive, *CH25H* ^+^, and T_reg_) across tissue groups. (D) Expression histogram of the T cell exhaustion gene *CTLA4* in each CD4^+^ cell subpopulation. (E) Expression specificity of *CH25H* in CD4^+^ cell subsets. (F) Heatmap of marker gene expression in CD4^+^ T cell subpopulations. Red signifies increased expression. (G) Immune infiltration analysis of *CH25H* in CD4^+^ cells. (H) Survival analysis of *CH25H* in GC: the left figure shows that the high expression of *CH25H* is positively correlated with the poor prognosis of GC (*p* = 0.0032). The right figure shows that high expression of *CH25H* is positively associated with poor prognosis of EGC (*p* = 0.044). (I) UMAP view of 10 CD8^+^ T cell clusters. (J) Proportions of each subset in the CD8^+^ T cluster. (K) Proportions of three representative CD8^+^ T cell subpopulations (T_EM_ CD8^+^, *GFPT2* ^+^ CD8^+^, and MAIT) across tissue groups. UMAP, uniform manifold approximation and projection.

We first focused on T cells. Further subclustering analyses revealed eight CD4^+^ T‐cell clusters: CD4‐proliferating‐T (*MKI67*); CD4‐T_eff_ (effector T, *CCL5*), a cytotoxic gene [16]; CD4‐T_EM_‐T (effector memory T, *IL7R*) [40]; CD4‐Regulatory‐T (T_reg_, *FOXP3* and *CTLA4*) [41]; CD4‐T_RM_ (tissue‐resident memory T, *CD69*) [42]; CD4‐*CXCL1*3‐T_FH_ (follicular helper T, *CXCL13*) [43]; CD4‐*CCR7*‐naive (*CCR7*), *CCR7*, and *SELL* are naive markers [16] (Figure 3A and Figure S5A,B). Among them, the proportions of T_reg_ and *CCR7* ^+^ naive subpopulations increased in both EGC and AGC, whereas *CH25H* ^+^ CD4 + T cells were significantly enriched in EGC (Figure 3B,C). T_reg_ cells are a special subset of CD4^+^ T cells that maintain immune tolerance by suppressing the immune response, whereas naive T cells can differentiate into T_reg_ and participate in immune regulation [44]. The exhaustion‐related genes (*PDCD1*, *LAG3*, *TIGIT*, *HAVCR2*, and *CTLA4*) are highly expressed in T_reg_ cells and *CH25H* ^+^ CD4^+^ T cells but are expressed at low levels in *CCR7* ^+^ naive T cells (Figure 3D and Figure S5E). These findings suggest that exhausted CD4^+^ cells may be the main mediators of immune escape in EGC and AGC. Notably, we identified the understudied *CH25H* ^+^ CD4^+^ subpopulation [45, 46, 47], which has high *FOXO1* and *TNFSF8*/*CD153* expression at the same time [48, 49] (Figure 3A and Table S6). We also observed stage dependence (CAG‐IM and EGC), with *CH25H* ^+^ CD4^+^ cells as the dominant population (Figure 3B). Moreover, the expression of *CH25H* in *CH25H* ^+^ CD4^+^ cells was significantly greater than that in other subgroups (Figure 3E,F). Immune infiltration analysis of STAD in public databases (TIMER 2.0) revealed that the expression of *CH25H* was positively correlated with the infiltration abundance of CD4^+^ T cells and CD4^+^ naive T cells (Figure 3G). Through the analysis of cell‒cell communication via CellChat, we found that the abovementioned CD4^+^ T‐cell subsets (T_reg_, *CCR7* ^+^ naive, and *CH25H* ^+^ CD4^+^ T cells) closely interact with the cancer‐precluster (Figure S5F,G). Survival analysis revealed that high *CH25H* expression was correlated with poor prognosis in patients with GC (OS, *p* = 0.0032, left) and EGC (TNM‐I, OS, *p* = 0.044, right) (Figure 3H). These findings suggest that *CH25H* may be a specific marker in EGC.

Among CD8^+^ T cells, we identified 10 clusters: early exhaustion CD8‐proliferating‐T (*MKI67*, *BIRC5*, *RRM2*) [50], CD8‐T_EM_ (effector memory T) (*GZMK*, HLA‐DRA, *HLA‐DRB1*) [40, 51], CD8‐*XCL1*‐T_RM_ (*XCL1*, *XCL2*, *CCL4L2*), CD8‐*ZNF683*‐T_RM_ (*ZNF683*, *PTPRCAP*, *ZYX*), CD8‐*CD160*‐IEL (*KLRC2*, *CD160*, *KLRK1*), CD8‐*GFPT2* (*GFPT2*, *AUTS2*, *ELL2*) [52], CD8‐*TNFSF14*‐Activated (*TNFSF14*, *LTB*, *RELB*), CD8‐Tc17 (*KLRB1*, *CCR6*, *CCL20*), CD8‐T_ex_ (*GZMB*, *IL7R*, *ARL4C*), and MAIT (mucosal‐associated invariant T; *MUC5AC*, *GAST*, *TFF1*) (Figure S5C,D, Figure 3I,J, and Table S6). We found that the proportions of CD8‐T_EM_ and *GFPT2* ^+^ CD8^+^ T cells increased, whereas the proportion of MAIT cells decreased with the occurrence of GC (Figure 3K). CD8‐T_EM_ cells highly express the cytotoxic genes *GZMK* and *MHC‐II* genes (*HLA‐DRA* and *HLA‐DRB1*), which are recognized as markers of T‐cell activation and are highly abundant with GC progression [40, 51]. CellChat analysis revealed that the exhaustion phenotype of CD4^+^ (T_reg_, *CH25H* ^+^, and *CCR7* ^+^ naive) and active CD8^+^ (T_EM_ and MAIT) cells was associated with significant intercellular communication with the cancer‐pre cells we identified (Figure S5F,G). These results indicate that CD4^+^ and CD8^+^ cells have diverse immune statuses in EGCs.

### B cells and monocytes are increased in EGC

We then explored the B/plasma cell subsets and generated 5 clusters of B cells and 7 clusters of plasma cells (Figure S6A). There was no significant difference or regularity in the proportion of plasma cells in all the samples. We found that the proportion of B cells, including all five subclusters, in EGC dramatically increased, which was very low in NAG samples. However, the proportion of B cells in advanced GC was lower than that in EGC (Figure S6B). The marker genes of all subclusters are shown in Figure S6C. Among them, B‐C5 highly expressed *TCL1A* (Figure S6C). We also found that *MS4A1*/*CD20* expression was increased in all B clusters (Figure S6C), and *MS4A1*/*CD20* expression was verified to be increased across cancers (including gastric cancer) [53]. Immune infiltration analysis revealed that *TCL1A* and *MS4A1* were positively correlated with Bn and B cells, respectively (Figure S6D).

We continued to characterize the heterogeneous myeloid cell subsets. In addition to mast cells, we identified seven myeloid cell states, including three clusters for dendritic cells (DCs) and two clusters for macrophages (M1 and M2), monocytes, and neutrophils (Figure S6E). M1 macrophages were increased in TNM stages II and III but decreased in TNM stage IV. The number of monocytes was increased in EGC but significantly decreased in TNM‐IV tumors. Interestingly, the neutrophils were absent in the NAG samples but appeared in subsequent stages (Figure S6F). The identification of the canonical markers for each subcluster is shown in Figure S6G. CellChat revealed strong intercellular communication between the myeloid subpopulation and cancer‐pre cells (Figure S6H). These results indicate that B cells and monocytes are increased in EGC and that the changes in B/plasma and myeloid cells during GC progression are complex.

### Fibroblasts remain stable in EGC

**FIGURE 4:**
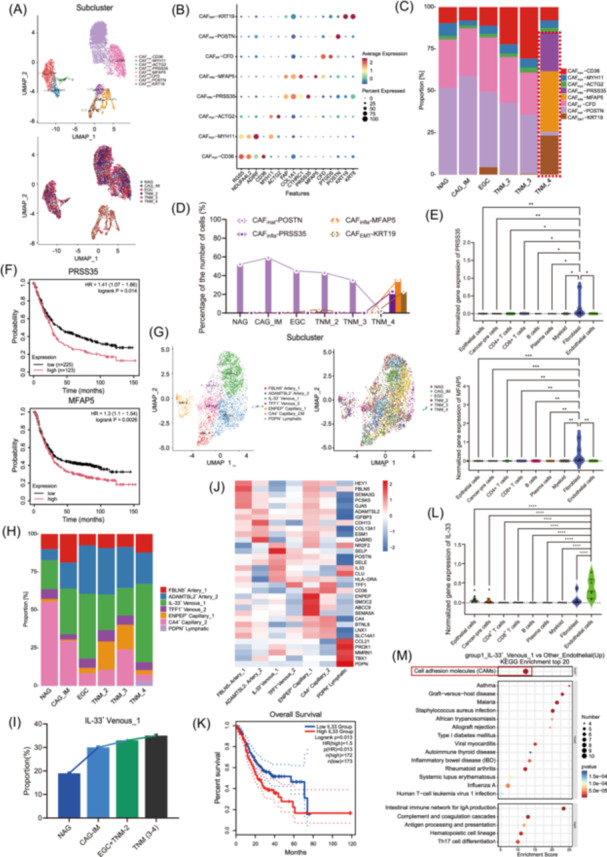
Stromal cell remodeling in GC progression. (A) UMAP showing the CAF subcluster. (B) Representative marker genes of the CAF subcluster. (C) Proportions of each subset of CAFs. (D) Proportions of four representative CAF subpopulations (CAF_mat_‐*POSTN*, CAF_infla_‐*PRSS35*, CAF_infla_‐*MFAP5*, and CAF_EMT_‐*KRT19*) across tissue groups. (E) Expression specificity of *PRSS35* and *MFAP5* in CAFs. (F) Survival analysis of *PRSS35* and *MFAP5* in GC. (G) UMAP view of 7 endothelial cell clusters. (H) Proportions of each subset of endothelial cell clusters. (I) Proportions of representative *IL‐33* ^+^ Venous‐1 subpopulations across tissue groups. (J) Heatmap of marker gene expression in endothelial cell subpopulations. Red signifies increased expression. (K) Survival analysis of *IL‐33* in GC. (L) Expression specificity of *IL‐33* in endothelial cells. (M) KEGG bubble map of DEGs between *IL‐33* ^+^ Venous‐1 and other endothelial cell subpopulations. p values were calculated via one‐way Kruskal‒Wallis rank‒sum tests. *p < 0.05; **p < 0.01; ***p < 0.001; ****p < 0.0001. CAFs, cancer‐associated fibroblasts; DEGs, differentially expressed genes; KEGG, Kyoto Encyclopedia of Genes and Genomes; UMAP, uniform manifold approximation and projection.

CAFs are known to influence tumor growth, migration, and invasion through the regulation of ECM components in various tumor types. However, the regulatory role and heterogeneous expression of specific CAFs in GC are still limited [54]. We divided fibroblasts (*THY1* and *COL1A2)* into 5 major clusters: matrix CAFs (CAF_mat_; *POSTN*), inflammatory CAFs (CAF_infla_; *PRSS35*, *MFAP5*) [55], adipocyte CAFs (CAF_adi_), myofibroblasts (CAF_myo_), and a newly defined subcluster with an epithelial‒mesenchymal transition phenotype (CAF_EMT_; *KRT19*) [56]. These clusters can be further divided into 8 subclusters (Figure 4A,B, and Figure S7A). The proportions of CAF_infla_ and CAF_EMT_ in AGC increased significantly, whereas CAF_mat_ decreased sharply (Figure S7B). The CAF_infla_‐*PRSS35*, CAF_infla_‐*MFAP5*, and CAF_EMT_‐*KRT19* subclusters were enriched in TNM‐IV, but the CAF_mat_‐*POSTN* subclusters were abundant, with the exception of TNM‐IV (Figure 4C,D). Moreover, there is intercellular communication between CAF_mat_, CAF_infla_, CAF_EMT_, and cancer‐pre (Figure S7C). Among the marker genes of the three subclusters (Figure S7D), *PRSS35* and *MFAP5* were specifically expressed in CAFs (Figure 4E) and associated with poor prognosis in GC patients (Figures 4F and S7E). In summary, the fibroblasts in EGCs remain stable and change in the advanced stage.

### The proportions of *IL‐33* ^+^ Venous‐1 and *ADAMTSL2* ^+^ Artery‐2 increased, whereas those of *CA4* ^+^ Capillary‐2 dramatically decreased in EGC

Tumor angiogenesis and endothelial dysfunction can affect tumor oxygenation, alter immune cell dynamics, and reduce drug penetration into tumors [57]. We performed subclustering analysis and identified the endothelial cell clusters *FBLN5* ^+^ Artery‐1, *ADAMTSL2* ^+^ Artery‐2, *IL‐33* ^+^ Venous‐1, *TFF1* ^+^ Venous‐2, *ENPEP* ^+^ Capillary‐1, *CA4* ^+^ Capillary‐2, and *PDPN* ^+^ Lymphatic (Figure 4G). The relative *IL‐33* ^+^ Venous‐1 and *ADAMTSL2* ^+^ Artery‐2 proportions gradually increased, whereas *CA4* ^+^ Capillary‐2 dramatically decreased with GC progression (Figure 4H,I). Lymphatic cells were detected in almost all the TNM‐IV samples (Figure 4H). The *IL‐33* ^+^ Venous‐1 subcluster was characterized by high expression of IL‐33 and cell adhesion genes, such as *MADCAM1* [58], *SELP*, and *SELE* [14, 59] (Figures 4J and S8A). We also compared specific marker genes in the *ADAMTSL2* ^+^ Artery‐2 (*ADAMTSL2*, *COL13A1*, *P2RY6*, and *ACOT7*) and *CA4* ^+^ Capillary‐2 (*CA4*) clusters. These genes have less tissue or cell specificity than Venous‐1 genes and have been less studied in gastric cancer (Figures 4J and S8A). Thus, we focused our research on the function of *IL‐33* ^+^ Venous‐1. We conducted a survival analysis of the *IL‐33* ^+^ Venous‐1 marker genes, and the results revealed that high expression of *IL‐33*, *MADCAM1*, *SELP*/*CD62P*, and *SELE*/*ELAM1* in GC was correlated with poor patient prognosis (Figures 4K and S8B). There are many high‐intensity cell interactions between *IL‐33* ^+^ Venous‐1 and *ENPEP* ^+a^ Capillary‐1. In addition, *IL‐33* ^+^ Venous‐1 is involved in cell communication with the cancer‐pre subcluster (Figure S8C). We then detected the specific expression of *IL‐33* in GC and found that the expression of *IL‐33* in endothelial cells was significantly greater than that in other cells (Figure 4L and Table S14). Moreover, *MADCAM1*, *SELP*, and *SELE* also increased in endothelial cells (Figure S8D). Next, we analyzed in depth the genetic differences between *IL‐33* ^+^ Venous‐1 and other endothelial cell subpopulations (Tables S15 and S16). GO and KEGG analyses revealed that the marker genes of this subcluster were enriched in immune response and cell adhesion pathways (Figures 4M, S8E, and Tables S17 and S18) [58, 60, 61]. Together, our findings revealed that the proportions of *IL‐33* ^+^ Venous‐1 and *ADAMTSL2* ^+^ Artery‐2 increased, whereas the proportion of *CA4* ^+^ Capillary‐2 dramatically decreased in EGC.

### IL‐33 enhances angiogenesis, and *IL‐33* ^+^ endothelial cells promote the growth of both EGC and AGC organoids *ex vitro* and *in vivo*

**FIGURE 5:**
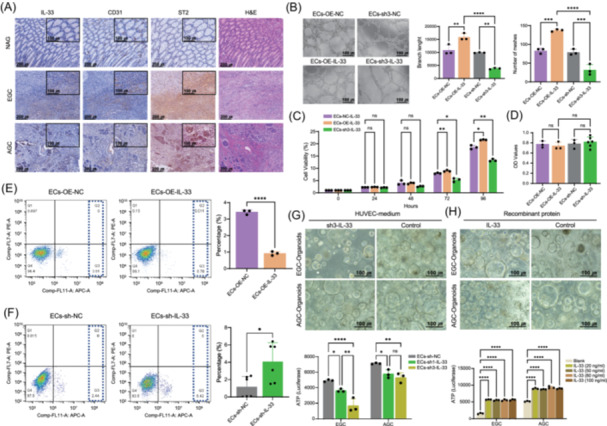
IL‐33 drives endothelial angiogenesis, and IL‐33^+^ ECs promote EGC and AGC growth ex vitro. (A) IHC staining of IL‐33, CD31, ST2, and H&E. Representative images of NAG, EGC, and AGC tissues are shown. Scale bars, 100 and 200 µm. (B) Tube formation images and bar graphs of HUVECs with knockdown or overexpression of IL‐33. (C) Proliferation of HUVECs with IL‐33 knockdown or overexpression. (D) Adhesion ability of HUVECs with IL‐33 knockdown or overexpression. (E, F) Apoptosis of HUVECs with IL‐33 knockdown or overexpression. (G, H) Images and histograms of the effects of IL‐33 on the proliferative ability of organoids. EGC and AGC organoids were treated with the culture supernatant of HUVECs with knockdown or overexpression of IL‐33 and recombinant IL‐33, and the ATP of the organoids was detected. *p* values were calculated via Student's *t*‐test. ns, not significant; *p < 0.05; **p < 0.01; ***p < 0.001; ****p < 0.0001. AGC, advanced gastric cancer; ATP, adenosine triphosphate; ECs, endothelial cells; EGC, early gastric cancer; H&E, hematoxylin‐eosin staining; HUVECs, human umbilical vein endothelial cells; IHC, immunohistochemistry.

Immunohistochemical (IHC) staining was conducted to analyze IL‐33 protein expression in EGC samples. We found that IL‐33 was localized mainly in the cytoplasm and membrane of endothelial cells (colocalized with the endothelial marker CD31). The expression of IL‐33 in EGC and AGC was greater than that in NAG (Figure 5A). Notably, ST2 (IL1RL1), the receptor of IL‐33, was highly expressed in both endothelial cells and tumor cells, and its expression increased gradually with the progression of gastric cancer (Figure S9A and Table S14). This expression pattern indicates that endothelia‐derived IL‐33 might affect the behavior of both cell types. We subsequently transfected human umbilical vein endothelial cells (HUVECs) with a lentivirus to knockdown or overexpress IL‐33 (Figure S9B,C). Ex vitro tube formation experiments revealed that the overexpression of IL‐33 increased branch length, the number of branch points, the number of meshes, and capillary length. However, knocking down IL‐33 suppressed these parameters (Figure 5B and Figure S9D). Furthermore, we found that IL‐33 promoted the growth of HUVECs (Figure 5C) and reduced apoptosis (Figure 5E,F). However, IL‐33 did not affect adhesion to the extracellular matrix (Figure 5D). We then generated organoids from clinical EGC (3 cases) and AGC (3 cases) samples to determine the role of IL‐33 in tumor cells (Figure S9E). The conditional medium from IL‐33‐knockdown HUVECs suppressed the growth of both EGC and AGC organoids (Figure 5G). Similarly, the recombinant IL‐33 protein facilitated the growth of both EGC and AGC organoids (Figure 5H).

**FIGURE 6:**
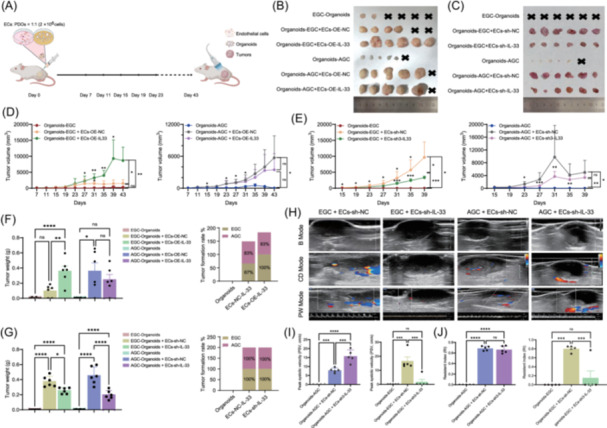
IL‐33^+^ ECs promote EGC and AGC angiogenesis and growth *in vivo*. (A) Diagram of a mouse subcutaneous transplanted tumor (EC cells and organoids). (B, C). Macroscopic images of the excised subcutaneous tumor mass upon sacrifice. No tumors were observed at the injection sites of the only EGC and AGC organoids (H&E‐confirmed inflammatory tissue). EC overexpression of IL‐33 promoted the growth of organoids (both EGC and AGC) subcutaneously (B). EC knockdown of IL‐33 inhibited the growth of subcutaneous organoids (both EGC and AGC) (both EGC and AGC) (C). (D, E) Tumor volume was monitored every four days, and tumor growth curves were drawn. EC‐OE‐NC had no significant effect on the growth of tumors compared with that of the only EGC organoid group (D). EC‐OE‐IL‐33 and EC‐OE‐NC had no significant effect on the growth of AGC organoids (E). (F, G) Tumor weights of the extracted subcutaneous tumors at the endpoint. ECs‐OE‐NC had no significant effect on the weight of tumors compared with the weight of tumors in the group with only EGC organoids. EC‐OE‐IL‐33 and EC‐OE‐NC had no significant effect on the weight of AGC organoids (F). The rate of subcutaneous tumor formation in the EC‐OE‐IL‐33 group of EGCs was greater than that in the EC‐OE‐NC group, and there was no difference in the other groups (G). (H) Ultrasound image of a mouse subcutaneous tumor. The EC‐sh‐IL‐33 group had more dark areas of fluid in the center of the subcutaneous tumors and a lower central blood supply. (I) Ultrasonic PSV detection of subcutaneous tumors *in vivo* at the endpoint. (J) Ultrasonic RI detection of subcutaneous tumors *in vivo* at the endpoint. The data represent the means ± SDs from six mice per group. ns, not significant; *p < 0.05; **p < 0.01; ***p < 0.001; ****p < 0.0001 versus empty vector control (two‐sided Wilcoxon rank‐sum test). AGC, advanced gastric cancer; B mode, brightness mode; CD mode, color Doppler mode; EGC, early gastric cancer; ECs, endothelial cells; H&E, hematoxylin‐eosin staining; NC, negative control; OE‐IL‐33, overexpression of IL‐33; PW mode, pulse‒wave Doppler mode; PSV, peak systolic velocity; RI, resistance index; sh‐IL‐33, knockdown of IL‐33.

We then tested the *in vivo* function of *IL‐33* ^+^ endothelial cells by coinjection of organoids and IL‐33‐manipulated HUVECs into SCID mice (Figure 6A). Subcutaneous neoplasms were identified via H&E and IHC (Figure S9F). The results revealed that the growth of EGC organoids was lower than that of AGC organoids without HUVECs, whereas endothelial cells increased the tumor‐formation capacity (Figure 6B,C). IL‐33‐overexpressing HUVECs further promoted the growth of both EGC and AGC organoids (Figure 6D,F), whereas IL‐33‐knockdown HUVECs exhibited the opposite effects (Figure 6E,G). Ultrasound revealed that the tumors that originated from the IL‐33‐knockdown group contained less blood supply and more necrosis, and vessels were mainly distributed in the tumor margin rather than the tumor core (Figure 6H). Moreover, the peak systolic velocity (PSV) and resistance index (RI) of the IL‐33‐knockdown group of EGC tumors were lower than those of the AGC group (Figure 6I,J), indicating that EGC was less malignant than AGC was. Taken together, these findings indicate that *IL‐33* ^+^ endothelial cells promote the growth of both EGC and AGC organoids ex vitro and *in vivo*. Furthermore, *IL‐33* ^+^ endothelial cells can increase vascular nourishment in tumors and increase the degree of tumor malignancy.

### IL‐33 upregulates the adhesion molecules PECAM1, CD34, and KRT17 in endothelial and tumor cells to promote tumor angiogenesis and growth

**FIGURE 7:**
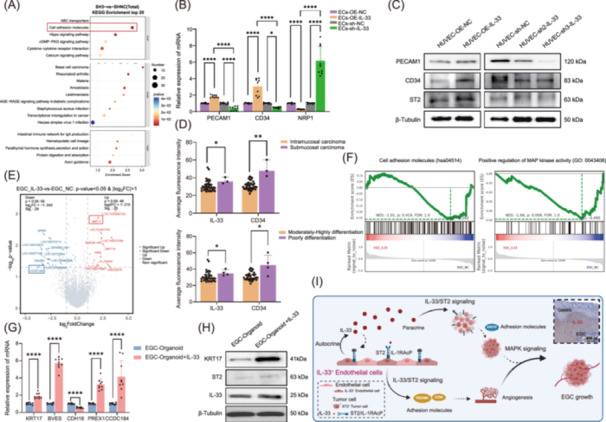
IL‐33 upregulates the expression of related adhesion molecules in ECs and EGC. (A) KEGG bubble map of DEGs between *IL‐33*‐knockdown ECs and negative control ECs. (B) RT‐qPCR was used to verify the DEGs in ECs after knockdown or overexpression of *IL‐33*. (C) WB was used to verify the gene expression of ECs after knockdown or overexpression of IL‐33. (D) ‌Tissue immunofluorescence showed that the expressions of IL‐33 (*p* = 0.045) and CD34 (*p* = 0.004) were higher in submucosal carcinoma than in intramucosal carcinoma. EGC with high expression of IL‐33 (*p* = 0.048) and CD34 (*p* = 0.011) was more malignant. (E) ‌DEG volcano plot of EGC organoids after the addition of IL‐33. (F) ‌GSEA revealed that DEGs were enriched in the cell adhesion and MAPK signaling pathways after ECs overexpressed IL‐33. (G) RT‒qPCR was used to verify the DEGs of EGC organoids after IL‐33 overexpression. (H) WB was used to verify the gene expression of organoids after IL‐33 overexpression. (I) Proposed a model of the mechanism in this study. IL‐33 activates the IL‐3/ST2 signaling pathway in an autocrine manner, enhances the expression of PECAM1 and CD34 in ECs, and promotes angiogenesis. High levels of IL‐33 also bind to ST2 in EGC organoids in a paracrine manner and activate the MAPK signaling pathway after the expression of KRT17 is upregulated, promoting the growth of EGC. IHC of IL‐33 at the boundary of normal gastric mucosa and EGC. Scale bar, 200 µm. The data are presented as the means ± SDs of three independent experiments. *p < 0.05; ****p < 0.0001 versus empty vector control (two‐sided Wilcoxon rank‐sum test). DEGs, differentially expressed genes; ECs, endothelial cells; EGC, early gastric cancer; GSEA, gene set enrichment analysis; KEGG, Kyoto Encyclopedia of Genes and Genomes; MAPK, mitogen‐activated protein kinase; NC, negative control; OE‒IL‒33, overexpressed IL‒33; RT‐qPCR, real‐time quantitative polymerase chain reaction; sh‒IL‒33, knocked down IL‒33; WB, western blot.

IL‐33/ST2 is a novel signaling pathway that contributes to tumorigenesis and plays a critical role in regulating angiogenesis and cancer progression in a variety of cancers. We subsequently used transcriptomic analysis to elucidate the mechanism of IL‐33 in endothelial cells and EGC organoids. First, the comparison of IL‐33‐knockdown HUVECs and control HUVECs (Figure S10A) revealed 908 upregulated genes and 407 downregulated genes (Figure S10B). These differentially expressed genes (DEGs) were enriched in cell adhesion molecules (Figure 7A). The significant DEGs associated with angiogenesis included *PECAM1*, *CD34*, and *NRP1*/*VEGF165R*. RT‐qPCR confirmed that *IL‐33* could upregulate *PECAM1* and *CD34* (Figure 7B and Table S19). IHC staining of subcutaneous xenografts revealed decreased PECAM1 expression in endothelial cells in the IL‐33‐knockdown group (Figure S9F). Western blot analysis revealed that the expression of PECAM1 and CD34 was positively correlated with the expression of IL‐33 in endothelial cells (Figure 7C). These results confirmed that IL‐33 facilitates angiogenesis by upregulating PECAM1 and CD34. To confirm the clinical correlation between IL‐33 and EGC, we performed tissue immunofluorescence on 41 clinical EGC samples (intramucosal carcinoma=38, submucous carcinoma=3). It was found that the EGC with high expression of IL‐33 and CD34 had deeper invasion and higher pathological malignancy (Figure 7D, Figure S10C, and Table S20. The clinical correlation analysis result is shown in Table 1.

**TABLE 1:** Correlation analysis of IL‐33 and CD34 expression and clinical parameters in EGC tissues.

Variable	Case	IL‐33	Z	*P*	CD34	Z	*P*
Depth of invasion							
Intramucosal	38 (92.68)	29.31 (26.51,32.84)	2.003	**0.045**	29.75 (27.61,33.48)	2.854	**0.004**
Submucosal	3 (7.32)	35.66 (33.67,40.61)			47.66 (41.91,60.29)		
Histopathological diagnosis							
Moderately‐Highly differentiation	37 (90.24)	29.29 (26.51,32.84)	1.977	**0.048**	29.70 (27.61,33.48)	2.548	**0.011**
Poorly differentiation	4 (9.76)	34.67 (32.92,38.14)			44.79 (36.67,53.98)		
Size (cm)							
<1 cm	26 (63.41)	29.31 (26.29,32.88)	0.92	0.357	31.53 (28.65,34.34)	−1.029	0.304
≥1 cm	15 (36.59)	30.44 (28.12,37.02)			29.34 (27.52,37.57)		
IHC (Ki67)							
<50%	18 (43.9)	29.00 (25.86,32.84)	−1.366	0.172	30.49 (28.65,35.74)	0.079	0.937
≥50%	23 (56.1)	30.15 (28.12,36.20)			31.62 (27.61,34.34)		

Abbreviation: EGC, early gastric cancer.

On the other hand, we compared the DEGs in the EGC organoid‐enriched IL‐33 groups with those in the control groups (Figure S10D) and identified 25 significantly upregulated genes and 29 downregulated genes in EGC (Figure S10E). A heatmap revealed that the addition of IL‐33 to EGC significantly upregulated the *KRT17* gene, whereas the *CCDC184*, *CDH16*, *PREX1*, and *BVES* genes were significantly downregulated (Figure 7E and Table S21). These genes were enriched mainly in cell adhesion molecule pathways and positive regulation of the mitogen‐activated protein (MAP) kinase activity pathway (Figure 7F). RT‒qPCR and WB verified that IL‐33 could upregulate KRT17 (Figure 7G,H). IHC staining of subcutaneous xenografts revealed decreased KRT17 expression in tumor cells in the IL‐33‐knockdown group (Figure S9F). Survival analysis revealed that high expression of *ST2*, *PECAM1*, *CD34*, and *KRT17* predicted poor prognosis in patients with gastric cancer (Figure S10F). These results showed that IL‐33 facilitated angiogenesis and tumor growth by upregulating KRT17. In summary, we found that IL‐33 promoted endothelial cell proliferation by upregulating PECAM1 and CD34 in an autocrine or paracrine manner; moreover, endothelial cell‐derived IL‐33 facilitated EGC growth by increasing KRT17 expression (Figure 7I).

## DISCUSSION

The prognosis of GC is dramatically improved by early diagnosis. EGC has long been considered a disease characterized by genomic and epigenetic alterations and chromosomal instability [62, 63, 64, 65]. With the development of scRNA‐seq technology, scholars have partially revealed the molecular characteristics of gastric cancer at the single‐cell level. In this study, we used scRNA‐seq of a large number of cells to reveal different cell compositions and states during the progression of gastric cancer. In addition, several different epithelial cell, T cell, and endothelial cell subpopulations associated with EGC were identified. Another notable aspect of our study was the use of a reliable preclinical organoid model of EGC combined with scRNA‐seq data and the finding that *IL‐33* ^+^ endothelial cells can promote angiogenesis and tumor proliferation in EGC by influencing adhesion molecules (PECAM1, CD34, and KRT17). Compared with published gastric cancer scRNA‐seq studies, our experimental design and analyses are differentiated by a large cell number (>180,000 cells, higher than most prior GC studies) and samples reflecting multiple clinical stages (*n* = 37 patients, 42 samples, including 10 EGC samples). The role of endothelial cell subsets in EGC was investigated and verified in patient‐derived organoids (PDOs) and animal models.

Tumors are in a complex microenvironment, and tumor‐infiltrating immune cells such as lymphocytes, myeloid cells, and B/plasma cells play a central role in the TME. Our results suggest that the greatest proportion differences in EGC‐associated T cells lie in _Tregs_, *CCR7* ^+^ naive T cells, *CH25H* ^+^ CD4^+^ T cells, and MAIT cells. Multiple studies have shown that cancer‐tissue‐infiltrating T cells are predominantly high‐effector T_reg_ (*FOXP3* ^+^ and *CTLA4* ^+^) in a majority of cancers and hinder effective tumor immunity in humans. They can be targeted to control physiological and pathological immune responses, for example, by depleting them to enhance tumor immunity or by expanding them to treat immunological diseases [41]. Previous studies have shown that CH25H (cholesterol 25‐hydroxylase) is associated with extracellular vesicles involved in tumor progression, cholesterol metabolism, and cellular immunity, but its role in T cells has not been clarified [66, 67]. We report that the *CH25H* ^+^ CD4^+^ cell subpopulation, which also expresses the CD4^+^ active markers *FOXO1* and *TNFSF8*, is increased in EGC. Interestingly, high proportions of exhausted genes were observed in T_reg_ and *CH25H* ^+^ CD4^+^ T cells in EGC, suggesting that T_reg_ and *CH25H* ^+^ CD4^+^ T cells are likely important T‐cell subsets for maintaining immune balance in the microenvironment of early‐stage cancer. In addition, *CCR7* ^+^ naive cells can be activated as central memory T cells and are present mainly in lymphoid tissue. The *CCL21*/*CCR7* axis regulates immune cell lymphatic migration and lymph node homing and participates in immune regulation [68]. We detected low expression of exhaustion genes in *CCR7* ^+^ naive cells, which may be related to their multidirectional differentiation characteristics. In EGC, *CCR7* ^+^ cells may mainly differentiate into memory T cells rather than into T_reg_ cells. MAIT CD8^+^ T cells constitute the only decreased T‐cell subcluster in EGC. MAITs are innate‐like T cells that recognize microbial metabolites through a semi‐invariant T‐cell receptor to activate their cytotoxic effector function [69]. Recent studies have shown that the number of MAIT cells is reduced in gastric, colorectal, and lung cancers [70]. A decrease in MAIT cells is closely related to the poor prognosis of patients with hepatocellular carcinoma [71] and is directly related to the pathological progression of *H. pylori*‐induced gastric cancer [72]. We also found that the proportions of CD8‐T_EM_ and *GFPT2* ^+^ CD8^+^ T cells in GC increased. T_EM_ cells express relatively high levels of receptors responsible for their migration to inflamed tissues and tumor lymph nodes. They receive dendritic cell MHC molecules to present antigens, thereby activating cellular immunity [73]. This finding is consistent with our results. Studies have shown that the infiltration density of CD8^+^ TEM cells increases in the core of primary colorectal cancer tumors and at the edge of liver metastases [74]. The expression of *GFPT2* in colorectal cancer was shown to be positively correlated with immunosuppressive cells, regulated immunosuppressive factors and T‐cell exhaustion, and increased in tumors [52]. However, no specific studies on *GFPT2* ^+^ CD8^+^ T cells have been reported. Although our study partially revealed the specific T‐cell immune composition of EGC, it could not reflect the spatial distribution characteristics of the cells due to the limitations of single‐cell technology.

Endothelial cells are the most important part of tumor vessels. In our datasets, we identified three endothelial cell subpopulations (*IL‐33* ^+^ Venous‐1, *ADAMTSL2* ^+^ Artery‐2, and *CA4* ^+^ Capillary‐2) that are characteristic of EGC and each expresses distinct subsets of novel endothelial cell markers. However, those genes generally lack specificity, such as *ADAMTSL2*, which is associated with cancer, angiogenesis and cell migration [75]; *COL13A1* is involved in cell‐matrix and cell‒cell adhesion interactions that are required for normal development, and existing studies have reported that it is produced by urothelial cancer cells and mainly maintains the tumor invasion phenotype [76]; *P2RY6* is a G‐protein‐coupled receptor that promotes tumor progression in colorectal cancer and lung cancer [77], and it has not been reported in gastric cancer; *ACOT7* is not expressed mainly in endothelial cells but is highly expressed in gastric cancer cells [78]; and carbonic anhydrase 4 (*CA4*) is involved mainly in maintaining the pH in cells, and some studies have shown that it is a drug that targets the tumor vasculature to inhibit angiogenesis in gastric cancer [79]. In contrast, Venous‐1 cell subsets express the specific endothelial cell genes *IL‐33*, *MADCAM1*, *SELP*, and *SELE*, which are closely associated with leukocyte adhesion, angiogenesis, and tumor progression [58, 60, 61].

IL‐33 exerts its functions in different ways depending on its location. As a nuclear factor, it can bind to the p65 subunit, which interfaces with the p65 subunit of nuclear factor‐B (NF‐κB) and reduces the expression of downstream pro‐inflammatory genes [80]. It can also bind to chromatin and histone proteins and affect gene expression by regulating DNA epigenetic inheritance [81]. Moreover, under conditions of cellular stress, such as infection and tumors, IL‐33 mainly binds to helper proteins (IL‐1RAcP) and receptors (ST2/IL1RL1) on the cell membrane, thereby activating cytokines or downstream signaling pathways [82]. The end effect of the IL‐33/ST2 signaling pathway depends on the ST2‐expressing cell types involved. In epithelial cells, various chemokines are produced, while in immune cells, cytokines such as IL‐4, IL‐5, and IL‐13 are released [23], which ultimately activate the pro‐inflammatory NF‐κB, MAP kinase p38, c‐Jun N‐terminal kinase, and extracellular signal‐regulated kinase (ERK) signaling pathways [83]. However, the role of IL‐33 in endothelial cells and tumors has been poorly investigated. In a variety of early solid cancers, IL‐33 is generally highly expressed [25]. In gastric cancer, IL‐33 and ST2 expression are higher in both intestinal metaplasia and GC tissues than in control tissues [22]. IL‐33/ST2 can lead to intestinal metaplasia and malignant transformation of gastric mucosa cells by regulating eosinophils [21] and M2 macrophages [19, 22]. IL‐33/ST2 can also regulate cell growth, proliferation, differentiation, migration, and apoptosis via the activation of *NF‐κB* [84], *PI3K*/*AKT* [85], *MAPKs* [86], and ERKs, such as *ERK1/2* [24]. In other cancers, IL‐33 is induced and activated early in the development of colorectal adenomas [87] and regulates the expression of the stemness genes *NANOG*, NOTC*H3*, and *OCT3/4* [26]. Furthermore, IL‐33 stimulates the release of proangiogenic factors in macrophages, such as VEGF and S100A8/9, which in turn activate endothelial cells to promote CRC angiogenesis [88]. In esophageal squamous cell carcinoma, a correlation has been observed between increased levels of IL‐33 and increased density of *FOXP3* ^+^ T_regs_ [89], which are thought to enhance immune escape [90]. This finding is consistent with the *FOXP3* ^+^ T_reg_ state we observed in EGC. Recent studies have shown that IL‐33/ST2 can regulate the progression of esophagitis to esophageal adenocarcinoma [27]. The role of IL‐33 in cancer is complex, considering that some studies have shown that it exerts an oncogenic role, whereas other studies have demonstrated the opposite [91]. Because of its important role in the inflammatory response, IL‐33 is expected to be a novel therapeutic target and predictor for cancer. Such as IL‐33 can be used as an early predictor of the efficacy of cetuximab in the treatment of colorectal cancer and the reduction of the toxicity of platinum‐based chemotherapy drugs to gastric cancer cells [91, 92, 93]. However, the application of IL‐33 in tumor therapy is still in the research stage. Several studies are exploring IL‐33 or its receptor ST2 as potential therapeutic targets. For example, humanized monoclonal antibodies against IL‐33 are developed for the treatment of related diseases (Application No. CN202010481666.9A). Due to the differences in the role of IL‐33 in different tumor types and microenvironments, its clinical use still needs to be carefully evaluated.

We found that *IL‐33* was more highly expressed in the Venous‐1 subcluster than in the other subclusters. These phenomena prompted us to explore the role of *IL‐33* ^+^ endothelial cells in EGC. On the one hand, IL‐33 can promote the proliferation and tubular formation of endothelial cells while reducing apoptosis. IL‐33 is expressed mainly in endothelial cells in clinical samples, while its receptor, ST2, is expressed mainly in tumor cells. The expression of IL‐33 was greater in EGC samples than in NAG and AGC samples. On the other hand, endotheliocyte‐derived IL‐33 promoted the proliferation of both EGC and AGC organoids ex vitro. Notably, *in vivo* mouse models have also demonstrated that IL‐33 produced by endothelial cells promotes xenograft growth and angiogenesis. We performed bulk transcriptome sequencing on samples after knocking down IL‐33 in endothelial cells and adding recombinant IL‐33 to EGC/AGC organoids. IL‐33 can upregulate the expression of endothelial adhesion molecules (PECAM1 and CD34) and organoid adhesion molecules (KRT17), thus promoting angiogenesis (cell‐to‐cell adhesion) and tumor proliferation. Previous studies have shown that IL‐33 affects cell‐matrix adhesion by promoting the activity of integrins [94], extracellular matrix components (such as collagen) [95], and matrix metalloproteinases [96], which is different from the intercellular adhesion of IL‐33 we found. And studies have confirmed that KRT17 can activate the ERK signaling pathway in bladder [97] and breast cancer [98] and promote cancer progression.

## CONCLUSION

As one of the most comprehensive single‐cell sequencing studies to date for EGC, our study provides a unique resource for generating novel biological insights into tumor cell types, subtype‐based TME compositions, and cell‒cell interactions in EGC. IL‐33 enhances the survival and angiogenesis of endothelial cells by upregulating the adhesion proteins PECAM1 and CD34. Endothelial‐derived IL‐33 could also promote the growth of EGC organoids through increasing KRT17 expression. Notably, we also found that high expression of IL‐33 was positively correlated with the depth of invasion and malignancy of EGC in the clinic. We anticipate future work to utilize combinatorial single‐cell approaches, including epigenetic, genetic, and transcriptional methods and spatial context, to enhance our understanding of the EGC architecture.

## METHODS

### Sample acquisition and tissue processing

Patients who were diagnosed with gastric adenocarcinoma and underwent surgical resection or endoscopy at the Second Affiliated Hospital of Chongqing Medical University, China, were enrolled after written informed consent was obtained. On‐table endoscopic biopsies or surgical resection samples were harvested. For surgical samples, matched normal gastric tissues from sites displaced at least several centimeters from the tumor were used. We follow strict aseptic procedures during specimen collection. The internal sample set contains 2 NAG samples, 5 CAG or IM lesions, and 6 EGC tissues, whereas the external sample set contains 3 NAG samples, 9 CAG or IM lesions, 4 EGC tissues, and 13 AGC samples. Tissues were collected in MACS tissue storage buffer (Miltenyi Biotec, DE) immediately after biopsy or resection and stored on ice. The samples were processed via enzymatic and mechanical dissociation via a human tumor dissociation kit and the Gentle MACS Octodissociator (Miltenyi Biotec, DE) following the manufacturer's instructions. Dissociated cells were passed through a MACS smart strainer (70 μm) and incubated with RBC lysis buffer for 10 min, followed by PBS neutralization. All centrifugation steps were carried out at 300 × *g* for 5 min. Dissociated cells were washed twice in PBS + 1% bovine serum albumin (BSA) and filtered through a 40‐μm smart strainer. Live‐cell counts were obtained via manual cell counting via a 1:1 trypan blue dilution. The cells were concentrated to 800–1200 live cells/μL and then processed for single‐cell analysis. The single‐cell processing program is strictly followed to avoid the impact of internal and external contamination on the results.

### Single‐Cell RNA Sequencing

The samples from each patient were processed in a single batch for library preparation. The Chromium Single‐Cell 3′ Library and Gel Bead Kit (10× Genomics) were used according to the manufacturer's protocols. Briefly, gel bead‐based emulsions (GEMs) were generated by combining barcoded single‐cell 3′ gel beads, cells, and partitioning oil. Ten barcoded, full‐length cDNAs generated from GEMs were amplified via PCR. The enriched libraries were enzymatically digested, size‐selected, and adaptor‐ligated for sequencing. The quantified libraries were sequenced on an Illumina Hiseq. 4000 sequencer. The MobiCube high‐throughput single‐cell 3′ transcriptome set V2.1 (PN‐S050200301) and the MobiNova‐100 microfluidic platform were used for scRNA‐seq. The single‐cell suspension was adjusted to an appropriate concentration (700–1200 cells/μL) and immediately loaded onto a chip to run on the MobiNova‐100 for microdroplet formation. Reverse transcription, cDNA amplification, and DNA library construction were performed according to the protocol. High‐throughput sequencing was performed in PE‐150 mode.

### Single‐Cell RNA‐seq data preprocessing

The FASTQ files were processed and aligned to the GRCh38 human reference genome using Cell Ranger software (version 8.0.1) from 10× Genomics, with unique molecular identifier (UMI) counts summarized for each barcode. The UMI count matrix was then analyzed using Seurat (version 4.0.0) R package. To remove low‐quality cells and likely multiplet captures, a set of criteria was used: 1) Cells were retained if their gene counts and UMI counts fell within the range of the mean ± 2 standard deviations; 2) Cells exhibiting a mitochondrial UMI percentage below 20% were retained; 3) Doublets were removed using DoubletFinder. Following these three filtering steps, the remaining cells were classified as high‐quality cells. scRNA‐seq data are commonly affected by technical artifacts known as “doublets,” which limit cell throughput and lead to spurious biological conclusions such as the discovery of mixed lineages. The DoubletFinder package (version 2.0.3) was subsequently used to identify potential doublets. After doublet cells were identified, they were removed from the data set. To obtain the normalized gene expression data, library size normalization was performed via the normalizeData function. Specifically, the global scaling normalization method “LogNormalize” normalized the gene expression measurements for each cell by the total expression, multiplied by a scaling factor (10,000 by default), and log‐transformed the results. The top 2000 highly variable genes were calculated via the Seurat function FindVariableGenes (mean.function = FastExpMean, dispersion.function = FastLogVMR). To remove batch effects from the single‐cell RNA sequencing data, mutual nearest neighbors (MNNs) were generated with the R package batchelor (version 1.6.3). Graph‐based clustering was performed to cluster cells according to their gene expression profile with the FindClusters function. The cells were visualized via a two‐dimensional uniform manifold approximation and projection (UMAP) algorithm with the RunUMAP function. The FindAllMarkers function (test.use = presto) was used to identify marker genes of each cluster. Differentially expressed genes were selected via the function FindMarkers (test.use = presto). A *p* value < 0.05 and |log_2_fold change| >1.2 were set as the thresholds for significantly differential expression. The top 20 differentially expressed genes (DEGs) for each cluster of the major cell types/lineages, including cancer‐pre, PMC‐like, CD4^+^ T, CD8^+^ T, myeloid, and stromal cells, are provided in Tables S13,15,22, and 23. Supplementary Table S6 lists the genes used for the various gene expression programs/modules. Combined with GO enrichment and KEGG pathway enrichment analyses of the DEGs were performed via R (version 4.0.3) on the basis of the hypergeometric distribution. The sequencing and bioinformatics analyses were performed by OE Biotech Co., Ltd.

### CellChat cell communication analysis

The CellChat (version 1.1.3) R package was used for cell‐to‐cell ligand–receptor interaction analysis. First, the standardized expression matrix is imported, and CellChat objects are created through the CellChat function. The default parameters were used to identify overexpressed genes, identify overexpressed interactions, and the preprocessing operations project data function. The computeCommunProb, filterCommunication (min.cells = 10) and computeCommunProbPathway functions were used to calculate potential ligand–receptor interactions. Finally, the intercellular communication network is aggregated by the aggregateNet function.

### Slingshot trajectory analysis

Slingshots can infer multiple developmental lineages from single‐cell gene expression data and sequence cells so that they appear as continuous processes with branches. This study uses the slingshot R package (version 1.8.0) for analysis. First, the reduced Seurat object is converted into a single‐cell experiment object by the as. SingleCellExperiment function. The cancer‐precell population is specified as the starting point (end point) to infer the cell development trajectory. A negative binomial generalized additive model (NB‐GAM) was used to fit the nonlinear function between gene expression and pseudotime values through the fitGAM function in the tradeSeq package (version 1.4.0). The associationTest function was used to select 100 genes with significant differences between gene expression and pseudotime values to draw heatmaps.

### CNV analysis

On the basis of the amount of gene expression in the single‐cell transcriptome data, CNV values for each region on the chromosome were assessed via the inferCNV (version 1.0.4) package (–cutoff 0.1). The cancer‐pre cells were selected as malignant cells, and all remaining cells were selected as normal cells. The genes were sequenced according to chromosome location, and 101 genes were used as sliding windows to calculate the average gene expression; normal cell expression was used as a control. The final CNV result file was generated after denoising. To avoid batch effects, we called the CNV for each sample separately on the gene‐expression matrix for all cells.

### Bulk RNA‐seq data analysis

To validate the function of *IL‐33* in PDOs and ECs, we analyzed its effects on genes at the transcriptional level (mRNAs) via RNA sequencing (RNA‐seq). For RNA isolation and library preparation, total RNA was extracted via the TRIzol reagent (Invitrogen) according to the manufacturer's protocol. RNA purity and quantification were evaluated via a NanoDrop 2000 spectrophotometer (Thermo Scientific). RNA integrity was assessed via an Agilent 2100 Bioanalyzer (Agilent Technologies). The libraries were subsequently constructed via the VAHTS Universal V6 RNA‐seq Library Prep Kit according to the manufacturer's instructions. Transcriptome sequencing and analysis were conducted by OE Biotech Co., Ltd.

mRNA sequencing analysis: The libraries were sequenced on an Illumina NovaSeq. 6000 platform, and 150 paired‐end reads were generated. Raw reads in fastq format were first processed via fastp (version 0.20.1), and the low‐quality reads were removed to obtain the clean reads. The clean reads were mapped to the reference genome via HISAT2 (version 2.1.0). The FPKM value of each gene was calculated, and the read count of each gene was obtained via HTSeq‐count. PCA was performed via R (version 4.0.3) to evaluate the biological duplication of samples. Differential expression analysis was performed via DESeq. 2 (version 1.22.2). A *Q* value < 0.05 and a fold change >2 or a fold change < 0.5 were set as the thresholds for significantly differentially expressed genes. Hierarchical cluster analysis of DEGs was performed via R (version 3.2.0) to demonstrate the expression patterns of genes in different groups and samples. A radar map of the top 30 genes was drawn to show the expression of upregulated or downregulated DEGs via the R packet ggradar. On the basis of the hypergeometric distribution, GO, KEGG pathway, Reactome and WikiPathways enrichment analyses of the DEGs were performed to screen the significantly enriched terms via R (version 3.2.0). R (version 3.2.0) was used to draw the column diagram, chord diagram and bubble diagram of the significantly enriched terms. Gene set enrichment analysis (GSEA) was performed via GSEA software. The analysis used a predefined gene set, and the genes were ranked according to the degree of differential expression in the two types of samples. Next, we tested whether the predefined gene set was enriched at the top or bottom of the ranking list.

### Generation and maintenance of gastric cancer PDOs

Human gastric tissues were biopsied from tumors and matched adjacent normal sites of patients during surgical intervention (Table S24). Briefly, the tissues were minced (1–3 mm), washed in phosphate‐buffered saline (PBS; Thermo Fisher Scientific) and digested in DPBS containing 1 mg/mL collagenase (Sigma‒Aldrich) and 2 mg/mL BSA (Sigma‒Aldrich, MO) for 20 min at 37°C. Once the mixture became cloudy, the digested tissues were passed through 30 μm filters (Miltenyi Biotec, DE). Filtered cells were pelleted at 300 × *g* for 5 min, resuspended in Matrigel (Corning Life Sciences), and seeded into multiwell plates (Thermo Fisher Scientific). Cultures were maintained in custom gastric PDO culture medium (#K2179‐GC, BioGenous, China) at 37°C in 5% CO_2_ and monitored daily for organoid generation. The culture medium in each well was replaced with fresh medium on alternate days. PDOs were passaged once every 7 to 10 days at a 1:3 ratio. The median establishment time to the respective passage at the time of sequencing was 17 weeks (range: 17–30 weeks; passage numbers: 9–11). Gastric organoids were harvested from gel matrices by washing briefly with PBS, incubating with trypsin‐EDTA at 37°C for up to 30 min, and pelleting at 300 × *g* for 5 min. The supernatants were discarded, and the cell pellets were washed twice with 10 mL of PBS each and filtered through cell strainers (mesh size: 30 μm). After centrifugation at 300 × *g* for 5 min, the supernatant was discarded, and the cells were washed with 1× PBS and then resuspended at ∼1000 cells/μL in 1× PBS containing 0.4% BSA.

### Endothelial cell culture experiments

Human umbilical vein endothelial cells (HUVECs) were purchased from the Cell Bank of the Chinese Academy of Sciences. HUVECs were cultured in endothelial cell medium (ECM) (#1001, SclenCell), which contained EGCS, 10% FBS (Gibco), 100 units/mL penicillin, and 100 μg/mL streptomycin (Beyotime, China), in a humidified incubator with 5% CO2 and 95% air at 37°C. The culture medium was changed every 2 days. The cells were passaged once every 2 days at a 1:2 ratio.

### Immunohistochemistry (IHC) assay

Formalin‐fixed paraffin‐embedded tissue composed of primary GAC tissues from a total of 388 patients who underwent total or subtotal gastrectomy was generated. Five‐millimeter‐thick tissue sections were deparaffinized in xylene, followed by dehydration in an ethanol series. The slides were incubated in H_2_O_2_ for 15 min at room temperature and subjected to high temperature and high pressure for antigen retrieval. Tris‐EDTA (pH = 8.0) was used as the retrieval buffer. The corresponding primary antibody was subjected to dropwise addition, followed by incubation at 4°C overnight, rinsing with PBS, and then dropwise addition of secondary antibody, avidin, and biotinylated HRP (#ZLI‐9036, ZSGB‐BIO). DAB (#ZLI‐9018, ZSGB‐BIO) solution was added to visualize the antibody binding, after which the sections were rinsed with distilled water, counterstained with hematoxylin, dehydrated with an ethanol gradient, and fixed with xylene and gelatin. Rabbit anti‐human IL‐33 polyclonal antibody (#12372‐1‐AP, Proteintech, 1:100, DE), rabbit anti‐human ST2 polyclonal antibody (#PRS3363, Merck, 1:100, DE), rabbit anti‐human CD31 polyclonal antibody (#11265‐1‐AP, Proteintech, 1:100, CA), rabbit anti‐human Ki67 polyclonal antibody (#28074‐1‐AP, Proteintech, 1:400, CA), rabbit anti‐human KRT17 monoclonal antibody (#26233, Cell Signaling Technology, 1:1200, MA), and secondary rabbit antibody (ZSGB‐BIO) were used.

### Lentiviral vector generation and transfection

HUVECs were grown in exponential growth conditions before lentiviral transformation. When the cells were subcultured and after they reached 50%–60% confluence, they were infected with the Ad‐control, Ad‐Not‐siRNA, or Ad‐Not‐oeRNA adenovirus at the optimal infectious titer according to the manufacturer's recommendations. The fluorescence intensity in each group of cells was recorded after 24 h, and the sorted cells were used for experiments and *in vivo* mouse studies.

### Quantitative real‐time PCR (qRT‐PCR) analysis

For total RNA extraction, when each cell line growing in a 6‐cm plate reached 70%–90% confluence, the medium was aspirated, and the cells were harvested via 500 μL–1 mL of TRIzol (Ambion) directly added to the plates. After vortexing vigorously and incubation at room temperature for 15 min, 200 mL of chloroform was added to each 1 mL of TRIzol, the mixture was vortexed vigorously again, and the mixture was incubated at room temperature for 15 min. The mixture was spun at maximum speed (15,000 rpm) for 10 min, the supernatant was transferred to a new tube, 2 volumes of ethanol were added to one volume of clear supernatant, the tube was gently vortexed, the tube was spun at maximum speed (12,000 rpm) for 10 min, the pellet was observed at the bottom, the mixture was gently washed with 70% ethanol, the mixture was spun at maximum speed for 5 min, and the supernatant was aspirated and air‐dried. The pellet was redissolved with an appropriate volume of 1× TE (pH 8.0) according to the size of the pellet. The total RNA concentration was measured with a Nanodrop 1000 instrument (Thermo Scientific). Reverse transcription and cDNA synthesis: We used NEB's LunaScript RT SuperMix Kit (E3010) following the manufacturer's protocol. Briefly, in a 20 mL reaction, LunaScript RT SuperMix (5×) (4 mL) was added to a tube with extracted total RNA, up to 1 mg. The 1st strand cDNA synthesis reaction was performed on a PCR machine with primers annealed at 25°C for 2 min, followed by cDNA synthesis at 55°C for 30 min and heat inactivation at 95°C for 1 min. The reactions were diluted with H_2_O to 200 mL in total volume. For the qPCR, a 20 mL total volume including 10 mL (2×) of SYBR Green Supermix from ABI (Applied Biosystems) with the addition of 2.5 mL of the above‐generated 1st strand cDNA was used, and PCR quantitation was performed on an Applied Biosystems QuantStudio 3 machine. The temperature was set at 95°C for 2 min, followed by 30 cycles of 95°C for 10 s and 60°C for 30 s. Analysis of gene expression was performed with *GAPDH* as the housekeeping gene. The data are presented via Microsoft Excel or GraphPad Prism. The reference gene primers and target gene primers used are listed in Table S25.

### Western blot analysis

Equal amounts of protein from each sample were separated via 10% to 12% sodium dodecyl sulfate‒polyacrylamide gel electrophoresis and electrotransferred onto polyvinylidene difluoride membranes. To prevent the nonspecific binding of antibodies, incubate the membrane with a blocking solution (commonly 5% nonfat milk or BSA in TBST) for 1 h at room temperature. The membranes were sequentially incubated with an IL‐33 primary antibody (Affinity Bio, dilution of 1 in 1000) or a GAPDH primary antibody (Proteintech, dilution of 1 in 3000) overnight and then with a horseradish peroxidase‐conjugated secondary antibody (anti‐rabbit or anti‐mouse, dilution of 1 in 3000 or dilution of 1 in 1000, Santa Cruz Biotechnology) for 1 h. Wash the membrane again to remove unbound secondary antibodies. The blots were developed with an enhanced chemiluminescence reagent (Beyotime) and quantified via densitometric scanning and analyses via a ChemiDoc™ XRS+ system (Bio‐Rad).

### Endothelial tube formation assay

The wells of the 96‐well microwell plates were coated with 50 µL of Matrigel, and the plates were placed at 37°C for solidification. HUVEC‐NC‐IL‐33, HUVEC‐sh‐IL‐33, and HUVEC‐OE‐IL‐33 cells were seeded in 96‐well plates at a density of 2 × 10^4^ cells per well. After incubation for another 12 h, the capillary‐like structures were observed with a light microscope.

### Cell adhesion experiment

A 96‐well plate was coated with 10 µg/mL fibronectin (FNA) for 1 h at room temperature. Two hundred microliters of heat‐denatured 1% BSA were added, and the plate was incubated at 37°C for 1 h. Medium immersion: Serum‐free medium was used to soak the orifice plate twice. A total of 5 × 10^4^ cells were seeded in a pretreated 96‐well plate. The cells were cultured in the incubator for 2 h, and the nonadherent cells were washed away with PBS three times. The number of cells in the 96‐well plate was detected via the CCK‐8 method.

### Cell apoptosis analysis

Cell apoptosis was measured with an Annexin V‐APC/PI apoptosis detection kit (#A214, Vazyme) according to the manufacturer's protocols. The cells were analyzed with a flow cytometer (Beckman Coulter). The data were analyzed via FlowJo software V.10.

### Cell proliferation assay

HUVEC‐NC‐IL‐33, HUVEC‐sh‐IL‐33, and HUVEC‐OE‐IL‐33 cells were seeded into 96‐well plates at a density of 5 × 10^4^/well. Three duplicate wells were set up for each sample. After the cells attached, a Cell Counting Kit‐8 (#HY‐K0301, MedChemExpress, NJ) was added to the medium at 100 µL/well, followed by incubation at 37°C for 2 h. The absorbance of each well was measured with a microplate reader at a wavelength of 460 nm [99].

### Conditioned medium generation

The HUVEC‐NC‐IL‐33, HUVEC‐sh‐IL‐33, and HUVEC‐OE‐IL‐33 cells were cultured in CO_2_ incubators for 2 days. These cells were maintained in ECM containing 10% FBS, 1% penicillin–streptomycin (v/v), and ECGS. When the cell density reached approximately 80%, the cells were washed with PBS and then cultured in serum‐free medium. Twenty‐four hours later, the CM was collected and centrifuged at 3000 rpm for 5 min to remove cell debris. The supernatant was concentrated five times with a Centricon‐10 concentrator (Macrosep, MAP010C38) and centrifuged for 20 min at 3000 rpm at 4°C. The media were collected and filtered through a 0.2 μm filter (Acrodisc, PAL‐4602). The supernatants were subsequently aliquoted and frozen at −80°C. The CM was used for further coculture experiments.

### Establishment of coculture systems

Gastric cancer PDOs were seeded in 96‐well plates. When the PDOs grew to a diameter of 50 µm, the original medium was discarded, and the mixed culture medium of each 96‐well plate was 100 µL with gastric cancer organoid medium (#B213152, BioGenous) and conditional medium. Recombinant IL‐33 (#HY‐P70475, MCE, CA) was diluted with gastric cancer organoid medium to concentrations of 20, 30, 50, 80, and 100 ng/mL. The culture mixture was changed every 2–3 days at a ratio of 1:1 for 24 h.

### Organoid viability ATP assay

Remove the 96‐well plates from the incubator and leave them at room temperature for 10 min to balance the plates to room temperature. Add the bioGenous^TM^ Organoid Viability ATP Assay Kit (#abs50059, Absin) reagent that has also been balanced to room temperature to the culture plate at a 1:1 volume ratio, absorb 100 μL of the test reagent, and add it to the organoid culture system containing 100 μL of the medium to be tested (without removing the matrix glue). The cells were fully lysed by linear vigorous oscillation (1000 rpm) with an enzyme labeler for 5 min, and the chemiluminescence values were read after 20 min at room temperature.

### In vivo tumorigenesis of IL‐33‐ECs in mice

Six‐week‐old SCID mice were randomly divided into 12 groups (EGC‐PDOs, EGC‐PDOs + ECs‐NC‐IL‐33, EGC‐PDOs + ECs‐OE‐IL‐33; AGC‐PDOs, AGC‐PDOs + ECs‐NC‐IL‐33, AGC‐PDOs + ECs‐OE‐IL‐33; EGC‐PDOs, EGC‐PDOs + ECs‐NC‐IL‐33, EGC‐PDO + ECs‐sh‐IL‐33; AGC‐PDOs, AGC‐PDOs + ECs‐NC‐IL‐33, AGC‐PDOs + ECs‐sh‐IL‐33). Each group received a subcutaneous injection of cells suspended in 50 µL of Matrigel into both lateral flanks of the mice. The ratio of PDOs to ECs was 1:1, and the number of cells in each injection was 2 × 10^6^. The tumor size was measured twice per week via a digital caliper, and the tumor volume was calculated with the following formula: volume = (width × length)^2^/2. The mice were killed 6 weeks after injection. All the tumors were collected and weighed.

### Subcutaneous ultrasound examination of the animals

Before ultrasound, the subcutaneous tumor area of each mouse was prepared, and the tumor location was fully exposed. The mice were anesthetized via a small animal anesthesia machine: the isoflurane concentration used to induce anesthesia was 3%, and the isoflurane concentration used to maintain anesthesia was 1.5%. After the skin surface of each mouse was coated with a coupling agent, a 30 MHz probe was selected, and the direction of the probe was adjusted. The anatomical structure of the subcutaneous tumor was recorded in B‐mode, the blood flow distribution of the subcutaneous tumor was recorded in CD‐mode, and the blood flow velocity and resistance index of the subcutaneous tumor were recorded in PV‐mode.

### Statistical analysis

All analyses were performed via GraphPad Prism (V.10.0), with statistical significance set at *p* < 0.05 adjusted for multiple testing. The Wilcoxon rank sum test was used to evaluate associations with continuous variables. Student's *t*‐test was used to evaluate associations with parametric continuous variables. Measurement data conforming to skew distribution were described by median and interquartile interval, and comparison between groups was performed by Mann–Whitney *U* test. Kaplan–Meier curves with log‐rank statistics were used to compare overall survival.

## AUTHOR CONTRIBUTIONS

**Zhihang Zhou, Song He, and Li Zhou:** Conceptualization. **Zhihang Zhou, Li Zhou, Mei Yang, Chao Deng, Manqiu Hu, Lili Zhang, Runmin Zha, and Yibo Tan:** Methodology. **Li Zhou and Mei Yang:** Software. **Chao Deng, Suhua Wu, Kewen Lai, Zhiji Chen, Qin Tang, Qingliang Wang, Lu Chen, and Yuanyuan Chen:** Investigation. **Li Zhou, Zhihang Zhou, Lu Chen, and Mei Yang:** Formal analysis. **Li Zhou and Zhihang Zhou:** Writing—original draft. **Li Zhou and Zhihang Zhou:** Writing—review and editing. **Li Zhou:** Visualization. **Zhihang Zhou, Song He, and Li Zhou:** Funding acquisition. **Zhihang Zhou and Song He:** Resources. **Zhihang Zhou and Song He:** Supervision. All authors have read the final manuscript and approved it for publication.

## CONFLICT OF INTEREST STATEMENT

The authors declare no conflicts of interest.

## ETHICS STATEMENT

This study was approved by the Ethics Committee of the Second Affiliated Hospital of Chongqing Medical University (Approval Number: 2021(115)). Primary PDOs and PDOs were isolated from patients who were diagnosed with gastric adenocarcinoma and who underwent surgical resection or endoscopic submucosal dissection (ESD) at The Second Affiliated Hospital of Chongqing Medical University. Patients were enrolled after written informed consent was obtained. Protocols were performed in accordance with the Declaration of Helsinki for Human Research. All animal experiments and procedures were approved by the Institutional Animal Care and Use Committee at Chongqing Medical University (IACUC‐CQMU‐2024‐0470).

## Supporting information


**Figure S1:** Endoscopic images and the hematoxylin and eosin (H&E) staining of new samples in this study.
**Figure S2:** Single‐cell atlas in gastric cancer (GC).
**Figure S3:** Epithelial cells subcluster of Cancer‐pre.
**Figure S4:** Epithelial cells subcluster of PCs (Proliferating cells), MSCs (Metaplastic stem‐like cells), and PMC like (Pit mucous like cells).
**Figure S5:** Tumor microenviroment remodeling in GC progression: immune cells (CD4^+^ and CD8^+^ T cells).
**Figure S6:** B cells and monocytes increased in early gastric cancer (EGC).
**Figure S7:** The fibroblasts remain stable in EGC.
**Figure S8:** Endothelial cells sub‐clusters.
**Figure S9:** Establishment of *IL‐33* ^+^ endothelial cells and organoids in GC.
**Figure S10:** *IL‐33* transcriptional level regulates differentially expressed genes in EGC and advanced gastric cancer (AGC).

**Table S1:** Clinical characteristics of newly samples used in scRNA‐seq study.
**Table S2 and S3:** Clinical characteristics of published samples used in scRNA‐seq study.
**Table S4:** Number of high‐quality cells.
**Table S5:** Number of cells for cluster.
**Table S6:** Metaclusters and Subclusters markers.
**Table S7:** Proportion of epithelial cell subtypes.
**Table S8:** Proportion of fibroblast subpopulation.
**Table S9:** Proportion of CD4^+^ T cell subsets.
**Table S10:** Proportion of CD8^+^ T cell subsets.
**Table S11:** Proportion of endothelial cell subtypes.
**Table S12:** Metaclusters Correlation.
**Table S13:** Top 20 differential expression genes of start vs end in Cancer‐pre‐Curve1 and Curve2 (up and down).
**Table S14:** Standardized data values for the amount of cell expression per subcluster.
**Table S15:** Top 20 differential expression genes of *IL‐33* ^+^ Venous‐1 vs Others endothelial cells (up and down).
**Table S16:** Top 70 differential expression genes of endothelial cells (up).
**Table S17:** GO enrichment in endothelial cells (up).
**Table S18:** KEGG enrichment in endothelial cells (up).
**Table S19:** Top differential expression genes of SH3 vs NC endothelial cells (up and down).
**Table S20:** Clinical characteristics of EGC patients undergoing immunofluorescence staining.
**Table S21:** Top differential expression genes of EGC‐IL‐33 vs EGC‐NC endothelial cells (up and down).
**Table S22:** Top 120 differential expression genes of B/plasma cells (up).
**Table S23:** Top 80 differential expression genes of fibroblast cells (up).
**Table S24:** Clinical characteristics of patient derived organoids.
**Table S25:** mRNA target sequences.

## Data Availability

The data that support the findings of this study are openly available in Genome Sequence Archive at https://ngdc.cncb.ac.cn/gsa-human, reference number HRA010477. The external GSE183904 (https://www.ncbi.nlm.nih.gov/geo/query/acc.cgi?acc=GSE183904) and GSE134520 (https://www.ncbi.nlm.nih.gov/geo/query/acc.cgi?acc=GSE134520) datasets are available at the National Institutes of Health (NIH). The raw sequence data reported in this paper have been deposited in the Genome Sequence Archive (Genomics, Proteomics & Bioinformatics 2021) in National Genomics Data Center (Nucleic Acids Res 2024), China National Center for Bioinformation/Beijing Institute of Genomics, Chinese Academy of Sciences (GSA‐Human: HRA010477). Due to the restrictions of human ethics and laws, GSA‐Human data cannot be publicly accessed. As a reasonable request, can through the following link (https://ngdc.cncb.ac.cn/search/specific?db=hra&q=HRA010477) to the corresponding author and Data Access Committee (DAC). And local laws, regulations, and rules should be followed, which includes submitting proposals to the DAC and signing data access agreements. Data can only be obtained after approval. Other relevant data supporting the main findings of this study can be obtained in the article and its supplementary information file. The data and scripts used are saved in GitHub (https://github.com/Zhouli33/EGC-paper-data-2025.git). Supplementary materials (figures, tables, graphical abstract, slides, videos, Chinese translated version, and update materials) may be found in the online DOI or iMeta Science http://www.imeta.science/.
