# Supplementary material for: Single‐cell sequencing reveals the role of IL‐33 + endothelial subsets in promoting early gastric cancer progression
Source: Imeta. 2025 Jun 5;4(4):e70050. doi: 10.1002/imt2.70050 (PMC12371260; doi:10.1002/imt2.70050)
Supplement: Supplementary file 1 — Figure S1: Endoscopic images and the hematoxylin and eosin (H&E) staining of new samples in this study. Figure S2: Single‐cell atlas in gastric cancer (GC). Figure S3: Epithelial cells subcluster of Cancer‐pre. Figure S4: Epithelial cells subcluster of PCs (Proliferating cells), MSCs (Metaplastic stem‐like cells), and PMC like (Pit mucous like cells). Figure S5: Tumor microenviroment remodeling in GC progression: immune cells (CD4+ and CD8+ T cells). Figure S6: B cells and monocytes increased in early gastric cancer (EGC). Figure S7: The fibroblasts remain stable in EGC. Figure S8: Endothelial cells sub‐clusters. Figure S9: Establishment of IL‐33 + endothelial cells and organoids in GC. Figure S10: IL‐33 transcriptional level regulates differentially expressed genes in EGC and advanced gastric cancer (AGC). [file IMT2-4-e70050-s002.docx]

**Supporting information to**

**Single-cell sequencing reveals the role of *IL-33*^+^ endothelial subsets in promoting early gastric cancer progression**

**Running title: A single-cell atlas revealed that *IL-33*^+^ endothelial cells can promote early gastric cancer.**

Li Zhou^1#^, Mei Yang^1,4#^, Chao Deng^1#^, Manqiu Hu^1^, Suhua Wu^1^, Kewen Lai^1^, Lili Zhang^1^, Zhiji Chen^1^, Qin Tang^1^, Qingliang Wang^2^, Lu Chen^2^, Runmin Zha^3^, Yuanyuan Chen^4^, Yibo Tan^1^, Song He^1*^, Zhihang Zhou^1*^

^1^Department of Gastroenterology, the Second Affiliated Hospital of Chongqing Medical University, Chongqing, China, 400010.

^2^Department of Pathology, the Second Affiliated Hospital of Chongqing Medical University, Chongqing, China, 400010.

^3^Department of Ultrasound, the Second Affiliated Hospital of Chongqing Medical University, Chongqing, China, 400010.

^4^Department of Gastroenterology, the Third People's Hospital of Chengdu, China, 610014.

^#^These authors contributed equally: Li Zhou, Mei Yang, and Chao Deng.

^*^Correspondence: [hedoctor65@cqmu.edu.cn](mailto:hedoctor65@cqmu.edu.cn) (Song He) and zhouzhihang@cqmu.edu.cn (Zhihang Zhou)

**Supplementary figures**

**Figure S1**


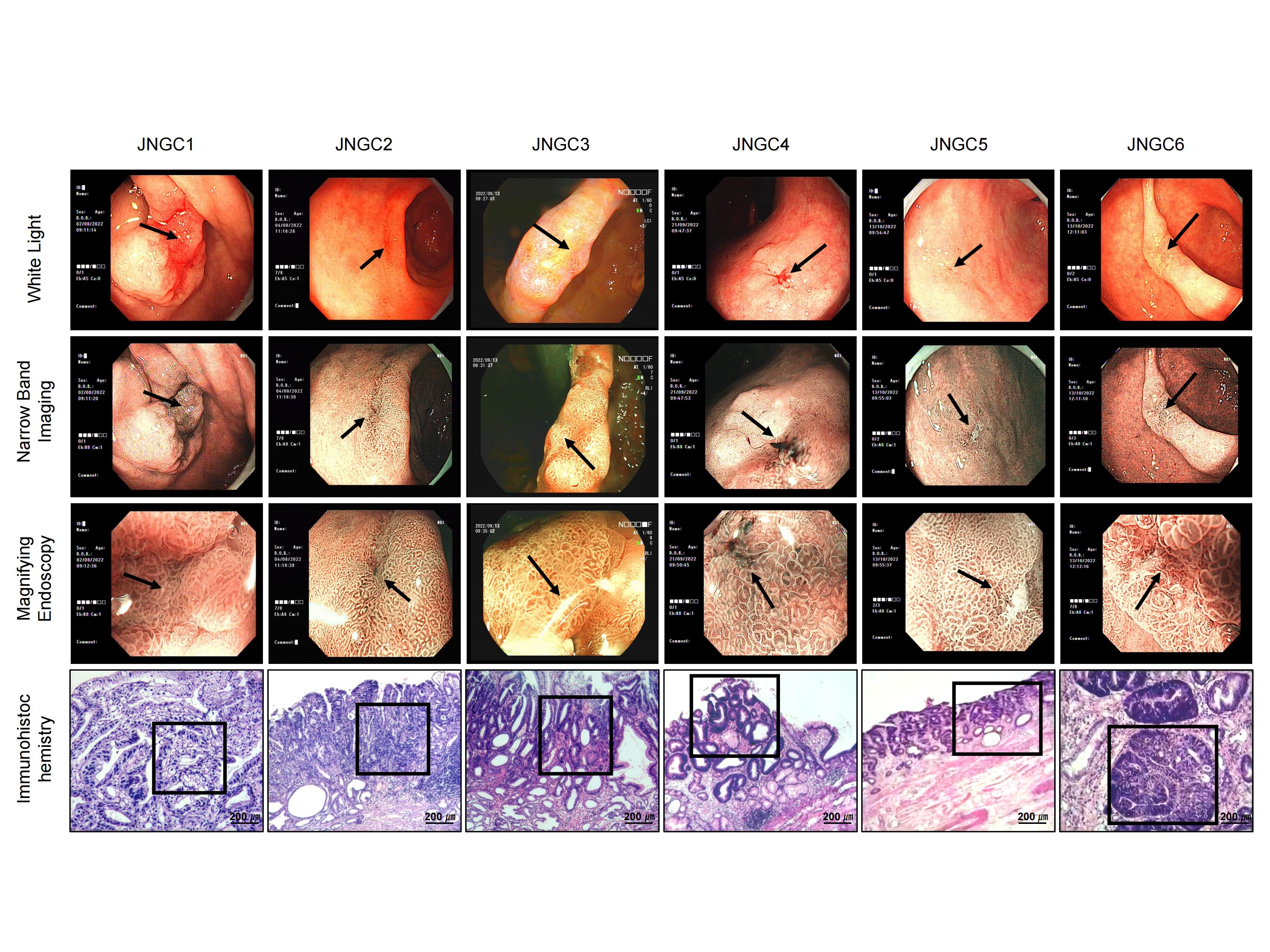


**Figure S1. Endoscopic images and the hematoxylin and eosin (H&E) staining of newly samples in this study.** Arrows show the sites of EGC biopsies. Mixed adenocarcinoma (JNGC1); Highly differentiated adenocarcinoma (JNGC2); Highly differentiated adenocarcinoma (JNGC3); In *situ* tubular adenocarcinoma (JNGC4); High-grade Intraepithelial Neoplasia (JNGC5); Highly differentiated adenocarcinoma (JNGC6). Scale bar, 200 μm.

**Figure S2**

**
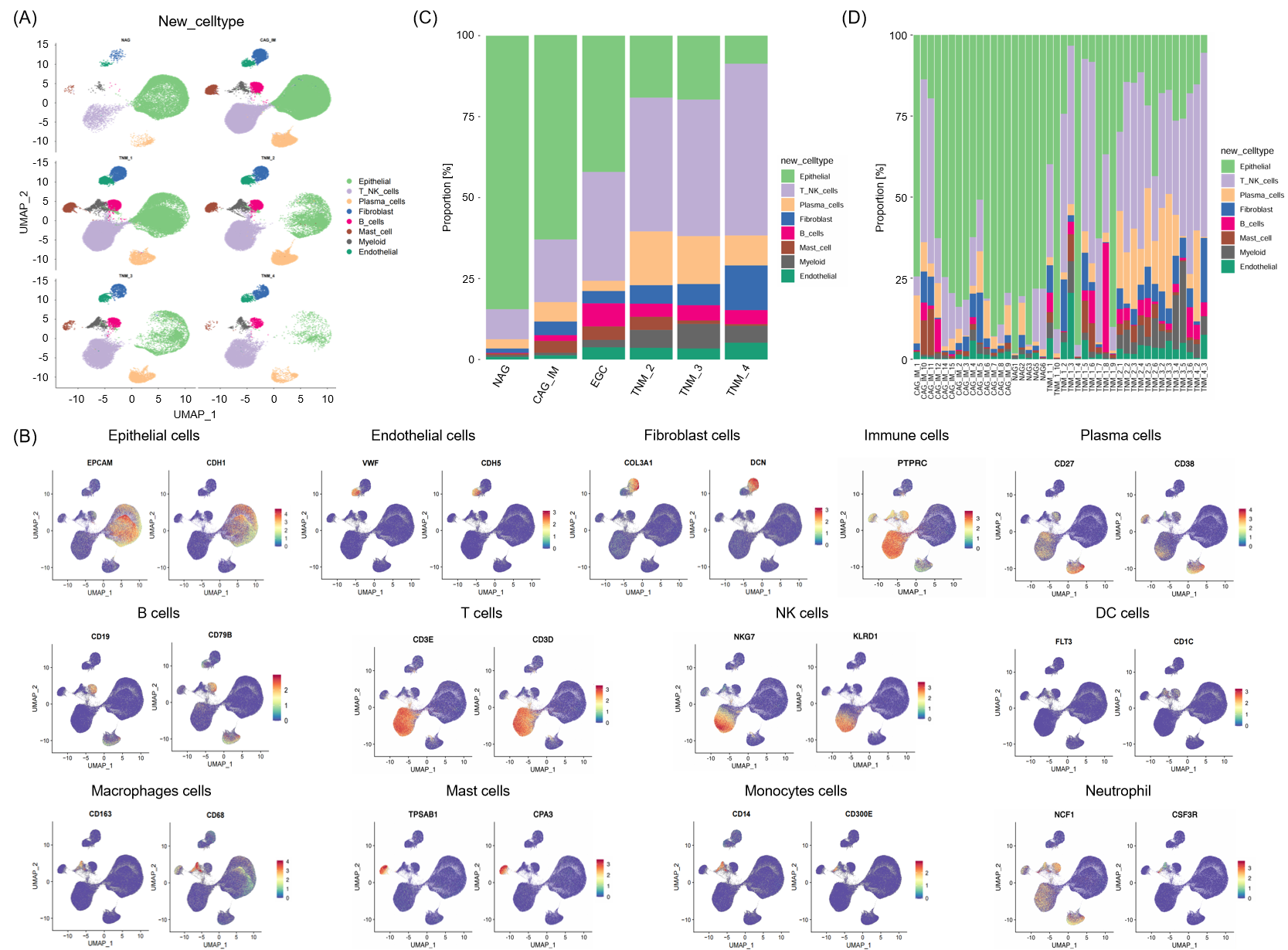
**

**Figure S2. Single-cell atlas in GC.** (A) A uniform manifold approximation and projection (UMAP) showing the classification of 184,426 single cells from NAG to AGC based on scRNA-seq data. (B) UMAP plots with color-coded marker genes indicating the expression levels (green to red) for major cell types. (C,D) The proportions of all meta-clusters across tissue groups.

**Figure S3**

**
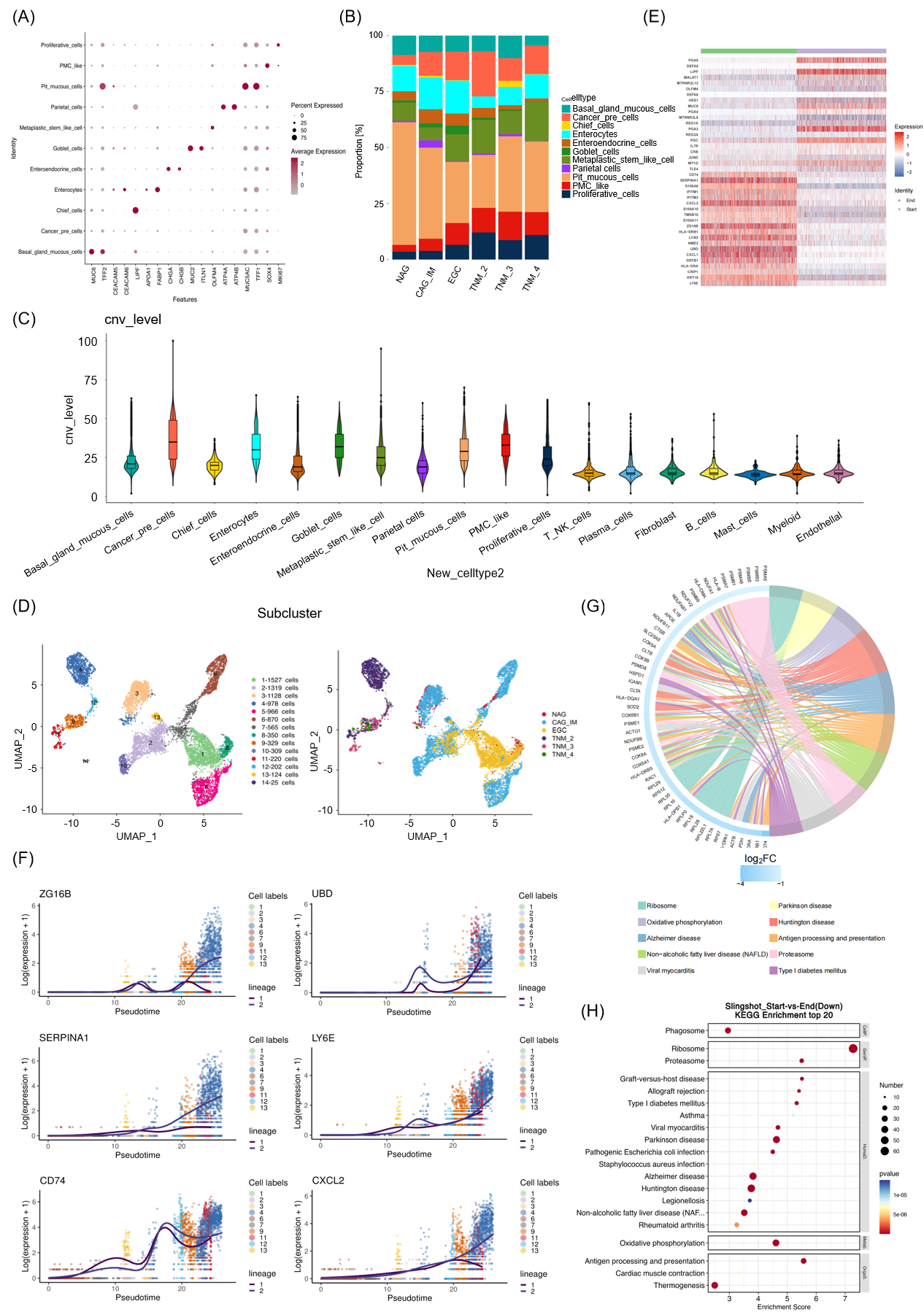
**

**Figure S3. Epithelial cells subcluster of Cancer-pre.** (A) Representative major metacluster marker genes. The size and color of circles show the percentage of cells expressing genes and average gene expression, respectively. (B) The proportions of epithelial cells across tissue groups. (C) Analysis of copy number variation (CNV) in epithelial cell subsets violin diagram. (D) UMAP of epithelial cells sub-cluster. (E) Heatmap representing the expression levels of different expression genes (DEGs) among cancer-pre Curve1 and Curve1 (start vs end) based on scRNA-seq data. (F) Expression dynamics of representative genes of cancer-pre (*ZG16B*, *UBD*, *CD74*, *SERPINA1*, *LY6E*, and *CXCL2*) in different tissues (color coded), along the pseudotime. (G) Chord diagram of significant pathways with DEGs (start vs end) enriched in cancer-pre-Curve1 and Curve2. (H) The top20 enriched pathways (KEGG) for down-regulated genes in cancer-pre (start vs end).

**Figure S4**

**
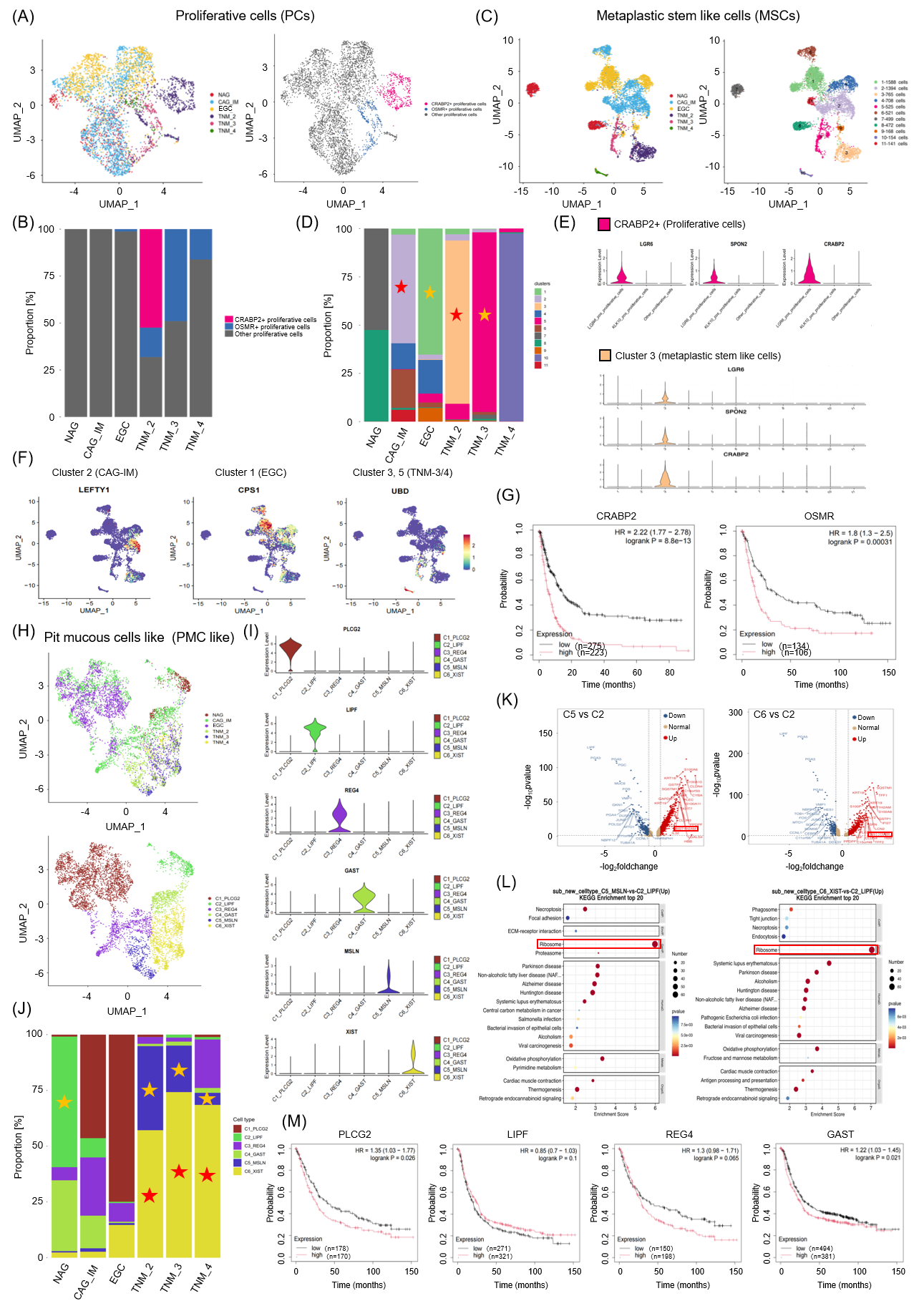
**

**Figure S4. Epithelial cells subcluster of PCs, MSCs, and PMC like.** (A) UMAP of proliferative cells (PCs) sub-cluster. (B) The proportions of PCs across tissue groups. (C) UMAP of metaplastic stem like cells (MSCs) sub-cluster. (D) The proportions of MSCs across tissue groups. (E) The violin plot showed that marker genes *LGR6*, *SPON2*, and *CRABP2* were highly expressed in both *CRABP2*^+^ PCs and C3 MSCs sub-clusters. (F) UMAP plots with color-coded marker genes indicating the expression levels (green to red) for C1 (CAG-IM), C2 (EGC), and C3/5 (TNM-III/IV) PCs. (G) Survival analysis (progression free survival, PFS) of representative genes (*CRABP2* and *OSMR*) in PCs. (H) UMAP of pit mucous cells like (PMC like) sub-cluster. (I) The violin plot indicating the expression profile of marker genes across multiple PMC like sub-clusters. (J) The proportions of PMC like across tissue groups. (K) ‌DEGs volcano plot of C5 (*MSLN*) vs C2 (*LIPF*) and C6 (*XIST*) vs C2 (*LIPF*) PMC like sub-populations. (L) The top20 enriched pathways (KEGG) for up-regulated genes in C5 (*MSLN*) vs C2 (*LIPF*) and C6 (*XIST*) vs C2 (*LIPF*). (M) Overall survival (OS) of marker genes *PLCG2* (C1), *LIPF* (C2), *REG4* (C3), and *GAST* (C4) in PMC like.

**Figure S5**

**
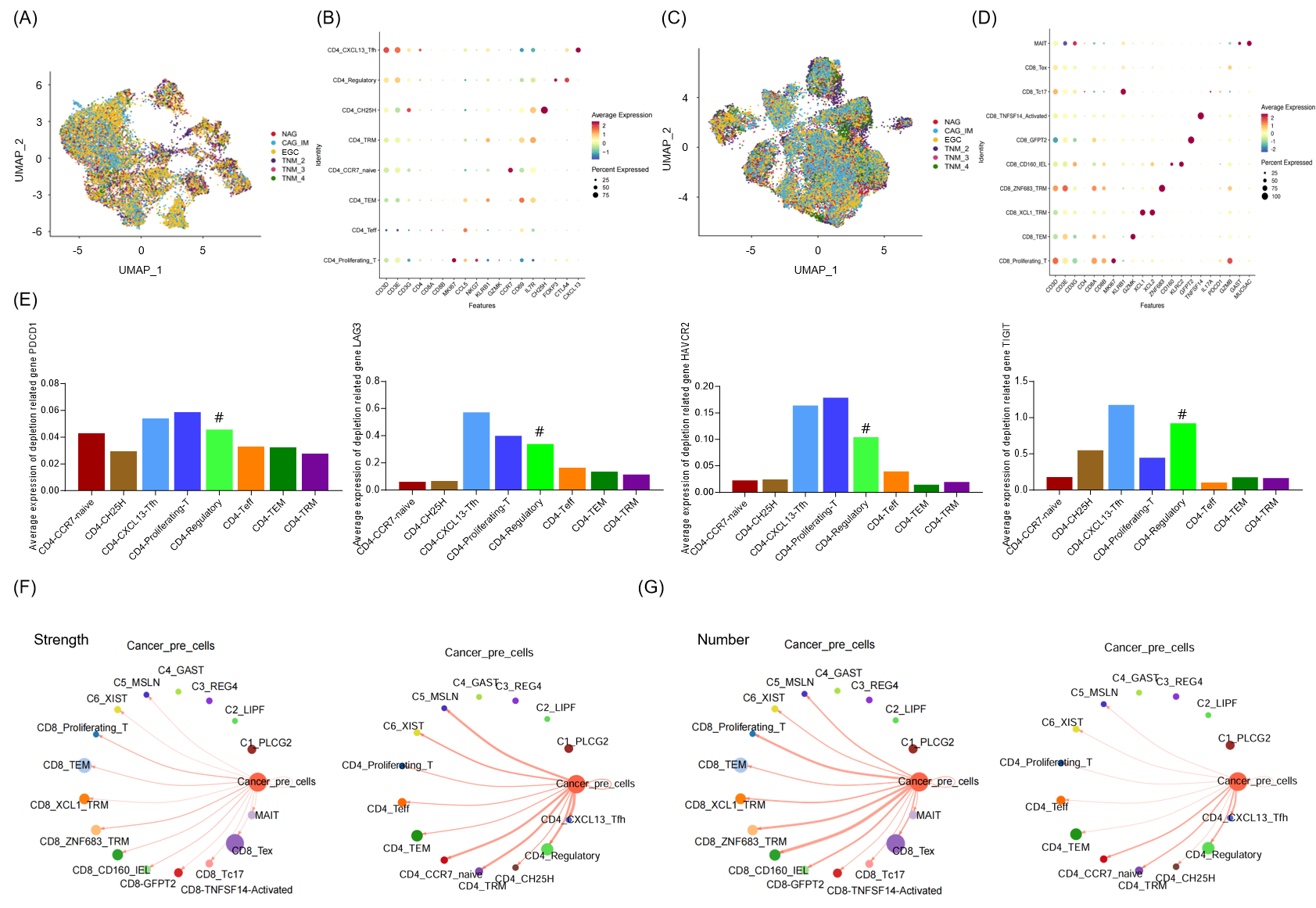
**

**Figure S5. Tumor microenviroment remodeling in GC progression: immue cells (CD4^+^ and CD8^+^ T cells).** (A) UMAP of CD4^+^ T cells sub-cluster. (B) Representative marker genes in CD4^+^ subclusters. The size and color of circles show the percentage of cells expressing genes and average gene expression, respectively. (C) UMAP of CD8+ T cells sub-cluster. (D) Representative marker genes in CD4^+^ subclusters. The size and color of circles show the percentage of cells expressing genes and average gene expression, respectively. (E) Expression histogram of T cell exhaustion gene *PDCD1*, *LAG3*, *HAVCR2*, and *TIGIT* in each CD4^+^ sub-populations. (F,G) Representative featured interaction networks (strength, F; number, G) identified through Cellchat analysis. The thickness of the flows indicates the relative communication probabilities. The colors of arrows indicate cell types that express outgoing signals.

**Figure S6**

**
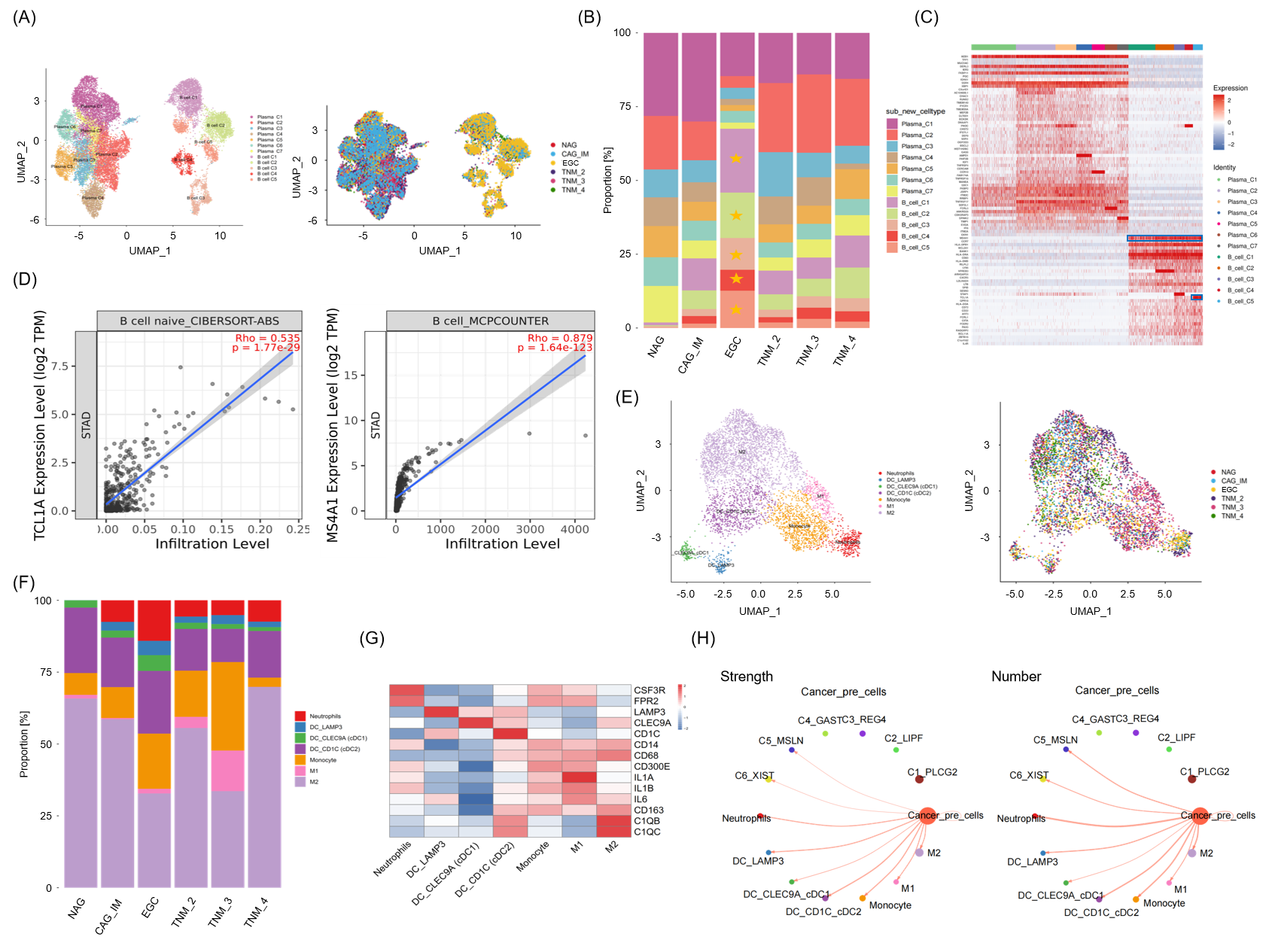
**

**Figure S6. B cells and monocytes increased in EGC.** (A) UMAP of B and plasma cells sub-cluster. (B) The proportions of B and plasma cells across tissue groups. (C) Heatmap representing the expression levels of different expression genes (DEGs) among B and plasma cells based on scRNA-seq data. (D) Immune infiltration analysis of *TCL1A* in naive B cells and *MS4A1* in B cells. (E) UMAP of monocytes sub-cluster. (F) The proportions of monocytes across tissue groups. (G) Heatmap of marker genes indicating the expression levels for monocytes subcluster. (H) Representative featured interaction networks (strength, left; number, right) identified through Cellchat analysis. The thickness of the flows indicates the relative communication probabilities. The colors of arrows indicate cell types that express outgoing signals.

**Figure S7**

**
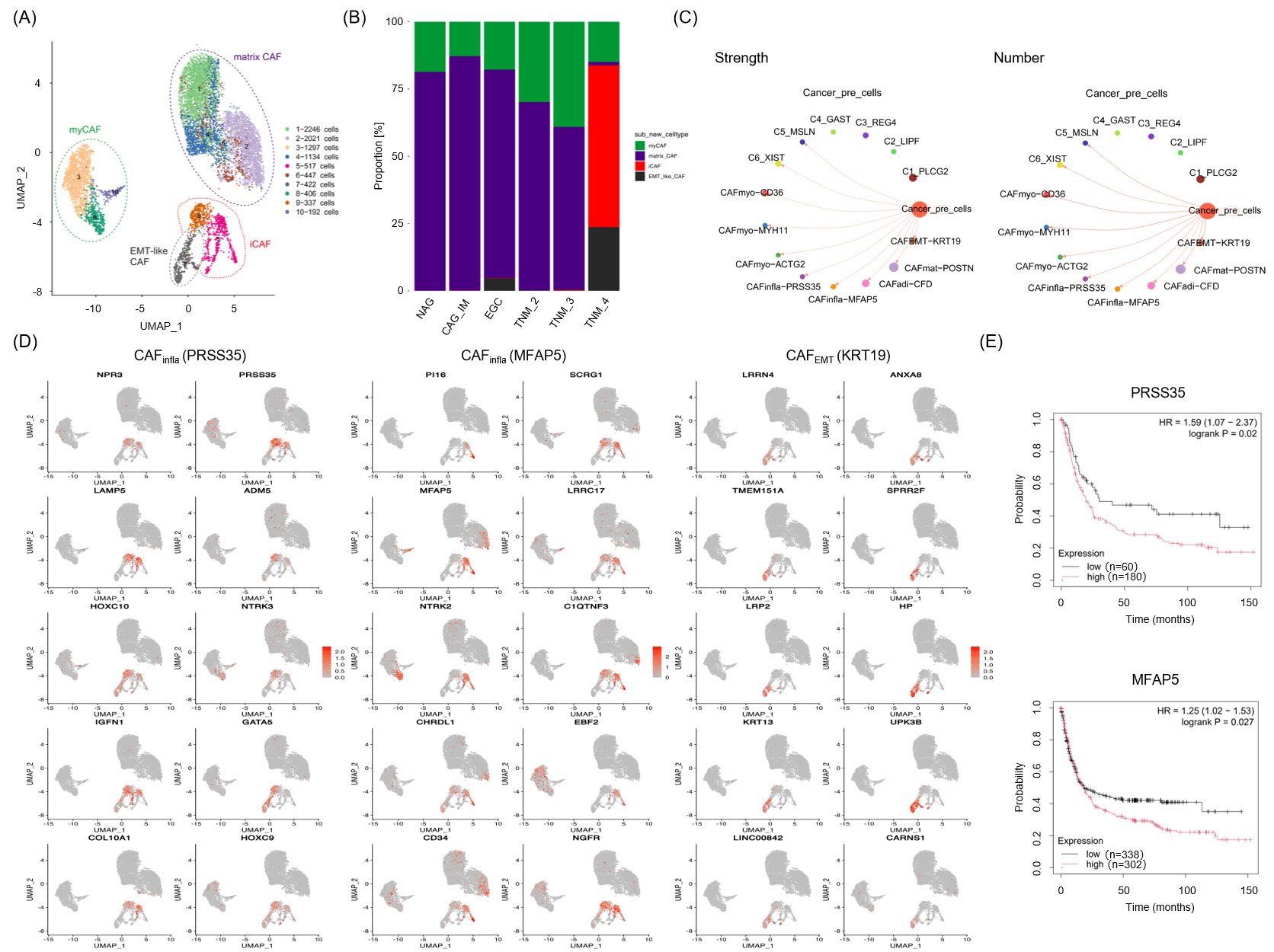
**

**Figure S7. The fibroblasts remain stable in EGC.** (A) UMAP of cancer-associated fibroblasts (CAFs) sub-cluster. (B) The proportions of CAFs across tissue groups. (C) Representative featured interaction networks (strength, left; number, right) identified through Cellchat analysis. (D) UMAP plots with color-coded marker genes indicating the expression levels (gray to red) for *PRSS35*^+^CAF_infla_, *MFAP5*^+^CAF_infla_, and *KRT19*^+^CAF_EMT_. (E) Overall survival (OS) of marker genes *PRSS35* (CAF_infla_) and *MFAP5* (CAF_infla_) in CAFs.

**Figure S8**


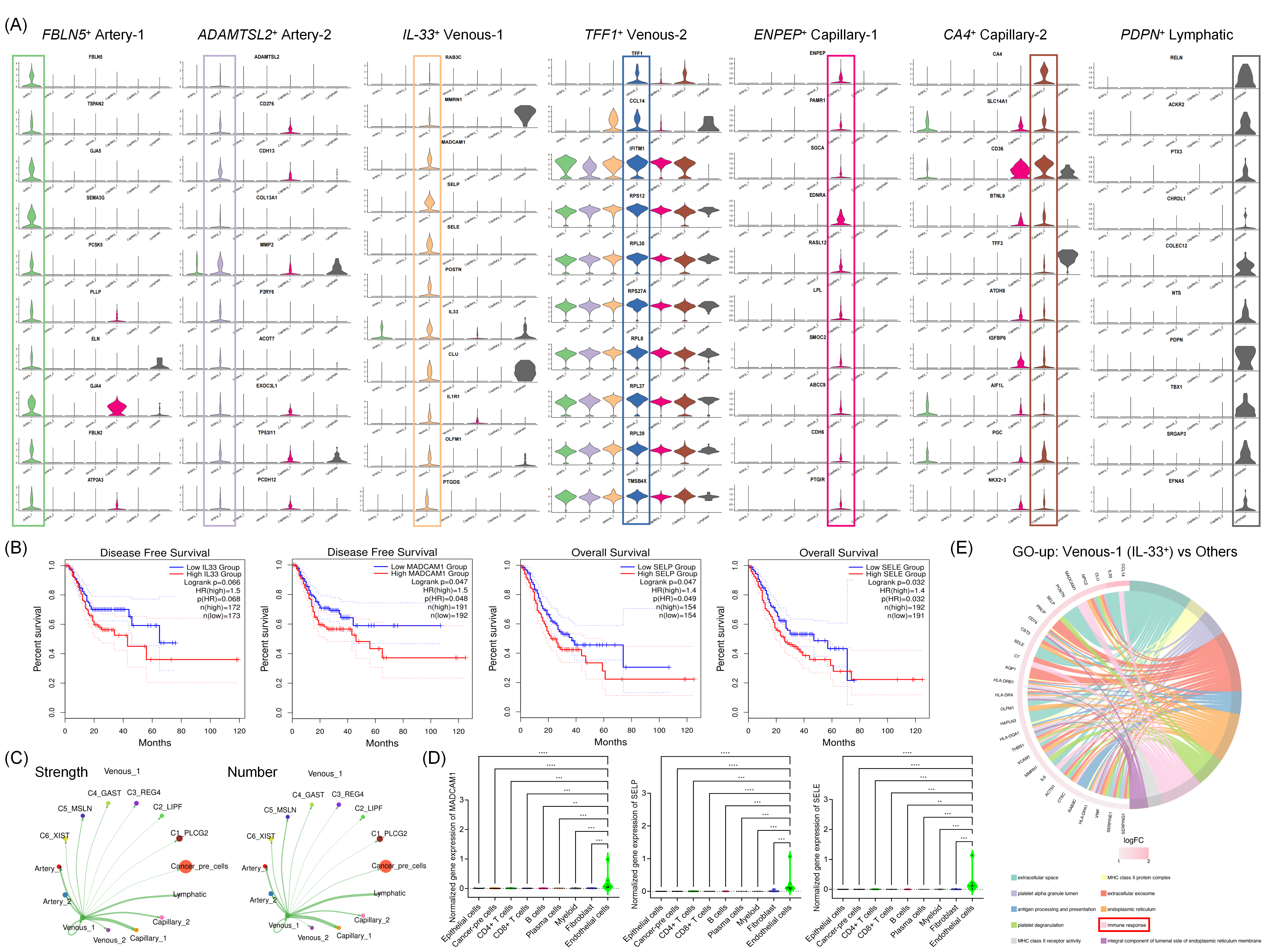


**Figure S8. Endothelial cells sub-clusters.** (A) The violin plot indicating the expression profile of top10 marker genes across multiple endothelial cell sub-clusters (*FBLN5*^+^ Artery-1, *ADAMTSL2*^+^ Artery-2, *IL-33*^+^ Venous-1, *TFF1*^+^ Venous-2, *ENPEP*^+^ Capillary-1, *CA4*^+^ Capillary-2, and *PDPN^+^* Lymphatic). (B) Disease free survival (DFS) of Venous-1 marker genes *IL-33*, Artery-2 marker genes *ADAMTSL2*, and Overall survival (OS) of *IL-33*^+^ Venous-1 marker genes *SELP* and *SELE*. (C) Representative featured interaction networks between endothelial cell and epithelial cell (strength, left; number, right) identified through Cellchat analysis. (D) Expression specificity of *MADCAM1*, *SELP*, and *SELE* in endothelial cells. (E) Chord diagram of significant up-regulation pathways with DEGs (*IL-33*^+^ Venous-1 vs others) in endothelial cells.

**Figure S9**

**
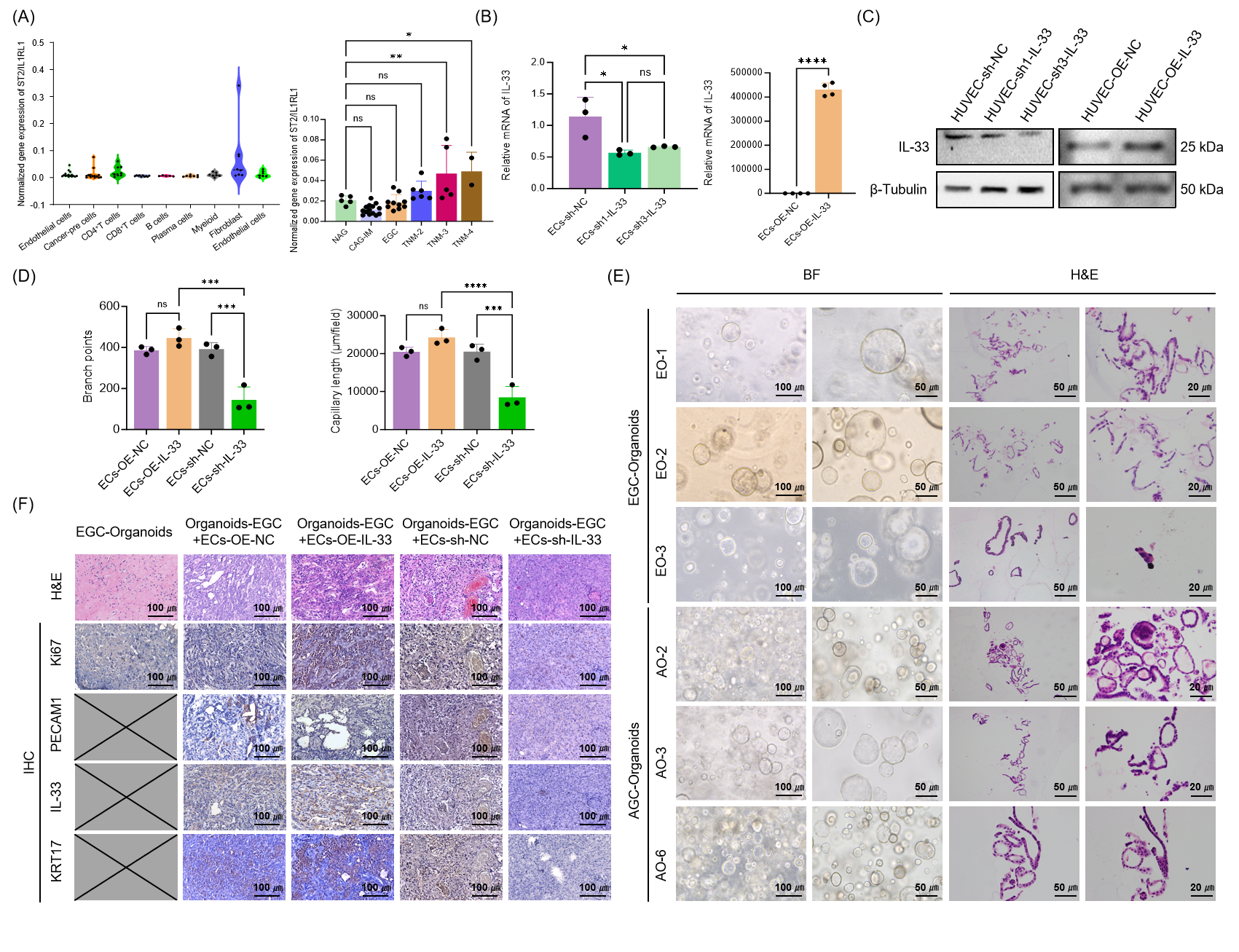
**

**Figure S9. Establishment of *IL-33*^+^ endothelial cells and organoids in GC.** (A) The violin plot showed the expression of ST2 genes across multiple meta-clusters and tissue groups. (B) HUVECs knockdown and overexpression of *IL-33* were verified by RT-qPCR. (C) HUVECs knockdown and overexpression of IL-33 were verified by western blot. (D) Tube formation images and bar graphs (branch points and capillary length) of HUVECs with knockdown or over-expression of IL-33. (E) White light and H&E staining images of organoids (3 EGC: EO-1, EO-2, EO-3;3 AGC: AO-1, AO-2, AO-3) culture. **f** IHC (Ki67, PECAM1, IL-33, and KRT17) and H&E images of mouse xenografts after subcutaneous injection of knockdown and over-expressing IL-33 HUVECs cells with EGC and AGC organoids. Scale bar, 20, 50, and 100 μm. *p* values were calculated by student’ s t-test. ns, no significance; **p* < 0.05; ***p* < 0.01; ****p* < 0.001; *****p* < 0.0001.

**Figure S10**

**
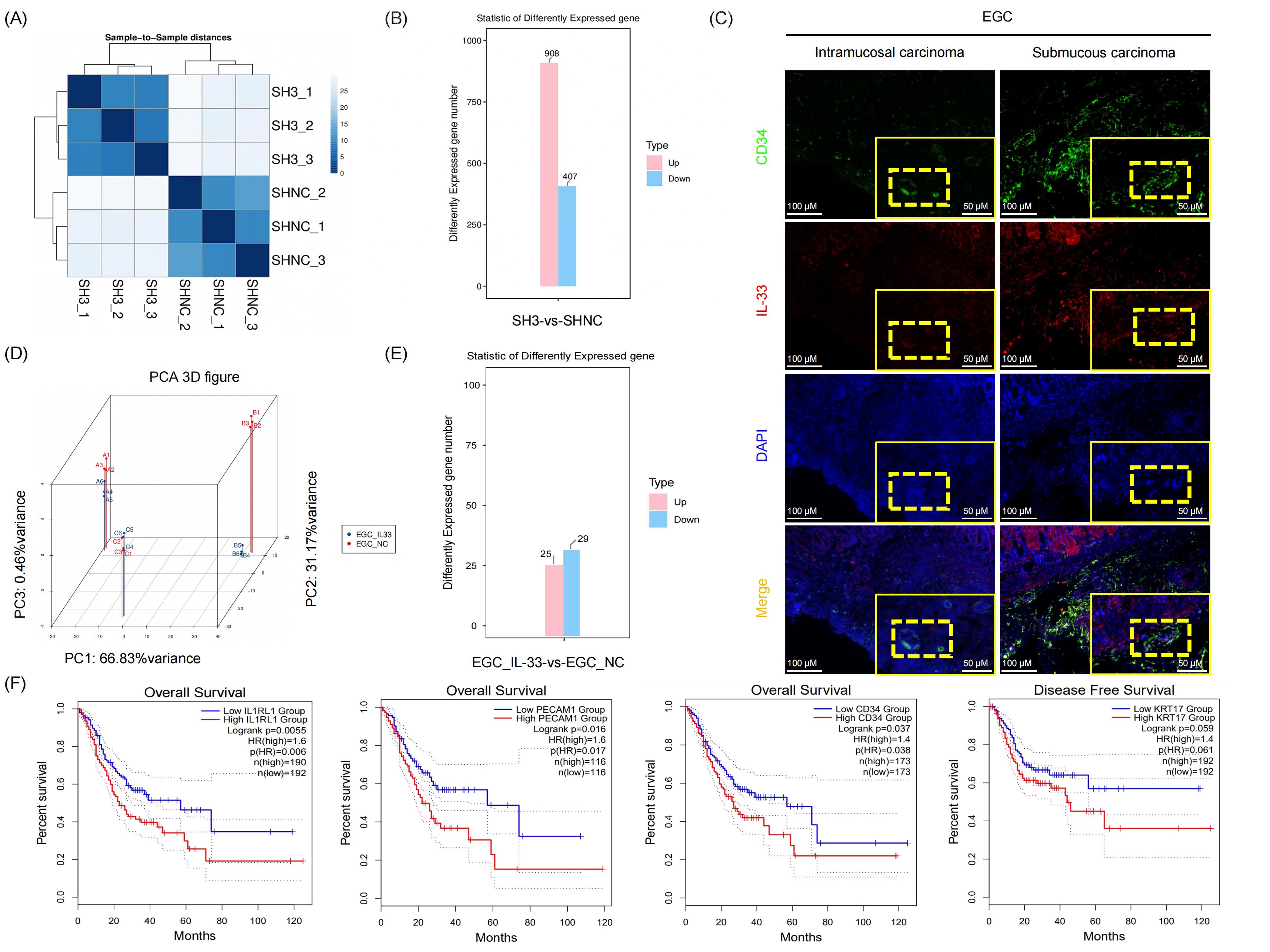
**

**Figure S10. *IL-33* transcriptional level regulates differentially expressed genes in EGC and AGC.** (A) Sample cluster (SHNC and SH3) analysis diagram. (B) Statistical histogram of differentially expressed genes between SHNC and SH3 (908 upregulation, 407 downregulation). (C) Immunofluorescence of a paraffin section of EGC (CD34 in green, IL-33 in red, DAPI in blue, and merge in yellow), with the vascular endothelium of intramucosal and submucosal carcinoma in the dashed box. The expression of IL-33 is low in the vascular endothelium of intramucosal carcinoma and high in the vascular endothelium of submucous carcinoma. (D) EGC-NC and EGC-IL-33 principal component analysis (PCA) diagram. (E) Statistical histogram of differentially expressed genes between EGC-NC and EGC-IL-33 (25 upregulation, 29 downregulation). (F) Overall survival (OS) of *IL-33/IL1RL1*, *PECAM1*, and *CD34*, disease free survival (DFS) of *KRT17*. SH3: HUVECs knockdown of IL-33; SHNC: control group; EGC-NC: only EGC organoid; EGC-IL-33: EGC organoid with recombinant IL-33.
